# Characterizing chain processes in visible light photoredox catalysis†
†Electronic supplementary information (ESI) available: Quantum yield measurements, luminescence quenching experiments, “light/dark” experiments, and time course data. See DOI: 10.1039/c5sc02185e


**DOI:** 10.1039/c5sc02185e

**Published:** 2015-07-07

**Authors:** Megan A. Cismesia, Tehshik P. Yoon

**Affiliations:** a Department of Chemistry , University of Wisconsin–Madison , 1101 University Avenue , Madison , Wisconsin 53706 , USA . Email: tyoon@chem.wisc.edu

## Abstract

The recognition that Ru(bpy)_3_^2+^ and similar visible light absorbing transition metal complexes can be photocatalysts for a variety of synthetically useful organic reactions has resulted in a recent resurgence of interest in photoredox catalysis. However, many of the critical mechanistic aspects of this class of reactions remain poorly understood. In particular, the degree to which visible light photoredox reactions involve radical chain processes has been a point of some disagreement that has not been subjected to systematic analysis. We have now performed quantum yield measurements to demonstrate that three representative, mechanistically distinct photoredox processes involve product-forming chain reactions. Moreover, we show that the combination of quantum yield and luminescence quenching experiments provides a rapid method to estimate the length of these chains. Together, these measurements constitute a robust, operationally facile strategy for characterizing chain processes in a wide range of visible light photoredox reactions.

## Abstract 2

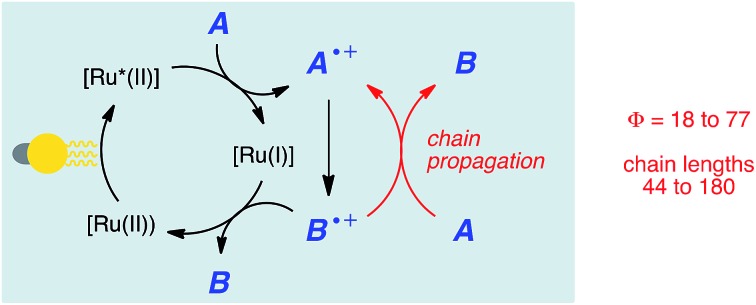
The combination of quantum yield and luminescence quenching measurements provides a method to rapidly characterize the occurrence of chain processes in a variety of photoredox reactions.

## Introduction

Over the past several years, a growing number of researchers have become interested in reactions that utilize Ru(bpy)_3_^2+^ and similar visible light-activated transition metal chromophores in synthetically useful photoredox reactions. Numerous recent studies have resulted in the development of an impressively diverse range of photocatalytic transformations;^1^ the applications of these reactions have ranged from natural product synthesis^2^ to late-stage pharmaceutical functionalization^3^ and polymer synthesis.^4^ Notably, the burst of renewed activity in visible light enabled photochemical synthesis has been accompanied by relatively little detailed mechanistic investigation.^5^ While the photophysical properties of Ru(bpy)_3_^2+^ and its analogues have been extensively studied and are well understood,^6^ only a handful of published reports have focused upon the equally important non-photochemical steps in photoredox transformations.^7^ Consequently, many salient mechanistic features of these reactions remain unclear.

**Scheme 1:**
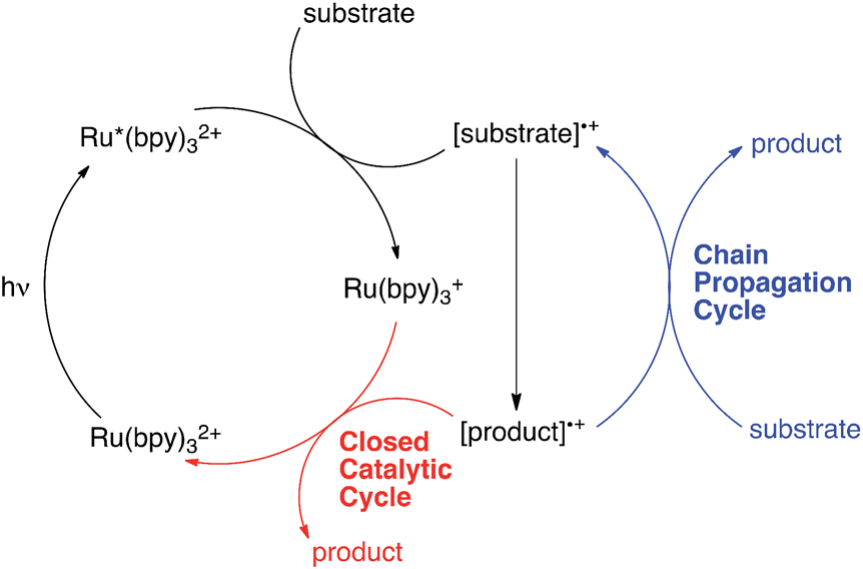
Generalized mechanism for oxidative photoredox reactions.

One area of significant disagreement has concerned the degree to which photoredox reactions involve chain processes. As a framework for this discussion, consider the generic mechanism for an oxidatively induced photoredox transformation depicted in Scheme 1. The essential details of the initial photochemical activation steps are not controversial: photoexcitation of Ru(bpy)_3_^2+^ produces a long-lived redox-active triplet state (Ru*(bpy)_3_^2+^) that can be reductively quenched by a wide range of organic substrates. The resulting radical cations ([substrate]˙^+^) are able to participate in a number of possible reaction manifolds, resulting in the formation of an open-shelled odd-electron product ([product]˙^+^). Many recent reports of photoredox transformations have posited that the generation of the final neutral product proceeds only *via* a closed catalytic loop (shown in red) involving reduction of this first-formed product by the reduced form of the photocatalyst (Ru(bpy)_3_^+^), which regenerates the photochemically active Ru(ii) state. Other researchers, however, have proposed that this class of reactions, like most other reactions of open-shell odd-electron reactive intermediates,^8^ are likely to involve chain mechanisms that operate in addition to the closed catalytic cycle.^9^ In this scenario, product formation would occur primarily *via* a chain propagation step (shown in blue) in which oxidized product radical cation interacts with another equivalent of neutral substrate, thereby generating the neutral product and another substrate radical cation by a single electron transfer process.^10^

The distinction between chain and non-chain mechanisms is an important one because the strategies appropriate for optimizing these two classes of reactions can differ significantly. For instance, if product is only formed by a closed catalytic loop, then optimization of the structural and electrochemical properties of the photocatalyst might be expected to strongly impact the efficiency of catalyst turnover. On the other hand, if chain reactions dominate product formation, then reaction variables that increase the rate of chain propagation or decrease the rate of chain termination should have a large effect even if they do not impact the activity of the catalyst itself.7a,^11^

One increasingly common study used to investigate the participation of chain processes is the so-called “light/dark” experiment, which examines the progress of a reaction in alternating periods of irradiation and darkness.^12^ The observation that productive reaction requires constant irradiation is commonly construed to mean that chain processes are either not occurring or are quite short. However, typical lifetimes for radical chain processes can often be on the second or sub-second timescale;^13^ the fact that conversion ceases during dark periods could also be consistent with chain processes that terminate faster than the timescale of the analytical measurement used, which may be several seconds or even minutes when an *ex situ* measurement is used.

Moreover, in a closed system, the concentration of [product]˙^+^ must be the same as that of the reduced photocatalyst (Ru(bpy)_3_^+^), which in turn can be no higher than the total initial concentration of photocatalyst. A non-chain catalytic cycle thus requires the encounter of two low-concentration reactive intermediates, which seems unlikely to produce the fast reaction times and low catalyst loadings reported in some of the most efficient photoredox methods. In addition, non-photochemical versions of many of these transformations are known and are widely accepted to involve chain processes.^14^ It therefore seems reasonable to consider the possibility that chain processes might be operative in a much wider range of photoredox reactions than is generally appreciated.

Quantum yield measurements provide a useful tool for identifying photochemical reactions that involve radical chains.^15^ The closed photoredox loop lacking chain propagation shown in Scheme 1 could exhibit a maximum theoretical quantum yield (*Φ*) of 1, which would indicate that every photon absorbed by the photocatalyst was producing one product molecule. This is the maximum value for this scenario: the occurrence of any non-productive photochemical processes such as phosphorescence, internal conversion, or back electron transfer would only decrease the observed quantum yield. Chain processes, on the other hand, could potentially provide multiple equivalents of product from each photon-induced initiation step. A reaction with *Φ* ≫ 1, therefore, could only be consistent with a product-forming chain.^16^

In this paper, we demonstrate that a combination of quantum yield and luminescence quenching measurements can provide a powerful method to study chain processes in synthetic photoredox reactions. We provide evidence that supports the involvement of chain propagation steps in three mechanistically diverse reactions involving radical cations, radical anions, and neutral radical intermediates. We further show that several important mechanistic features of these reactions are revealed using this analysis. Finally, we demonstrate that even reactions that unambiguously involve chain propagation steps can nevertheless require constant irradiation for product formation, and thus that “light/dark” experiments should not be used to definitively rule out the occurrence of chain processes in photoredox reactions.

## Results and discussion

### Photoinitiated radical cation reactions

We began our studies by investigating a photocatalytic radical cation Diels–Alder cycloaddition recently reported by our laboratory.^17^ This reaction was an attractive initial target for interrogation because a great deal is already known about the mechanisms of radical cation cycloaddition reactions, largely due to detailed investigations performed by Bauld^18^ and Ledwith.^19^ In particular, radical cation mediated [4 + 2] cycloaddition reactions can be conducted using sub-stoichiometric one-electron oxidants such as aminium radicals,^20^ and thus it is clear that these chemically initiated cycloadditions are chain reactions. It stands to reason, therefore, that photoinitiated versions of these reactions would likely also proceed through a chain mechanism.^21^

**Scheme 2:**
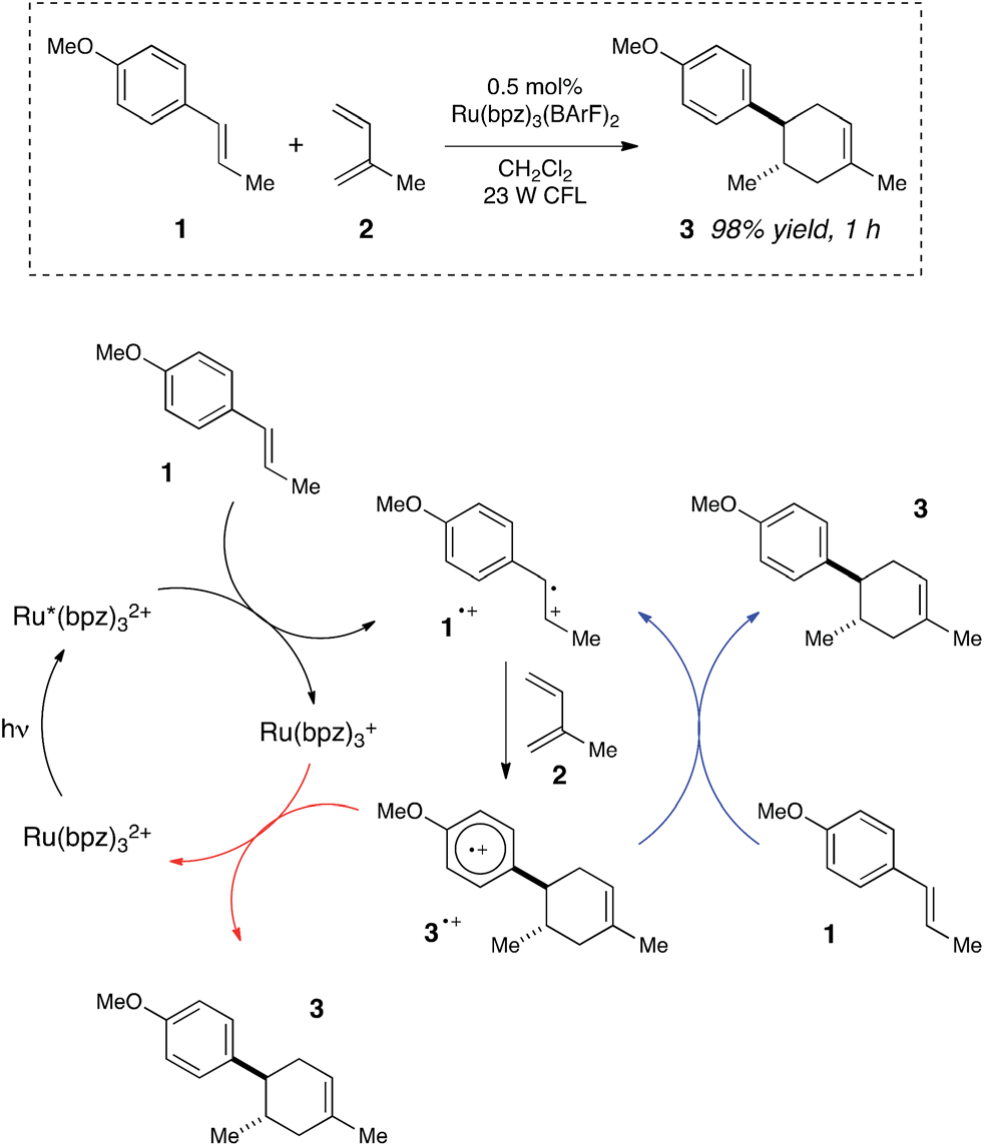
Chain and closed catalytic mechanisms for a photoredox radical cation Diels–Alder cycloaddition.

Our working model for the mechanism of the photoredox process is depicted in Scheme 2. The reaction is initiated upon reductive quenching of photoexcited Ru*(bpz)_3_^2+^ by anethole (**1**), which affords the alkene radical cation **1˙^+^** along with an equivalent quantity of the reduced catalyst Ru(bpz)_3_^+^. The alkene radical cation is activated towards [4 + 2] cycloaddition with a diene (**2**) to produce radical cation **3˙^+^** as the first-formed, open-shelled product of this reaction. In order to generate the neutral cycloadduct **3**, the product radical cation must be reduced by one electron. This can either occur *via* chain-terminating electron transfer from the reduced photocatalyst Ru(bpz)_3_^+^ (as shown in red), or *via* chain-propagating electron transfer from another equivalent of electron-rich alkene substrate **1** (blue).

To begin our investigations of chain propagation in the radical cation [4 + 2] cycloaddition, we calibrated its quantum yield against the photodecomposition of potassium ferrioxalate, a well-established chemical actinometer with known quantum efficiencies at multiple wavelengths.^22^ We selected 436 nm light for our experiments, a wavelength at which the Ru(bpz)_3_^2+^ photocatalyst absorbs strongly^23^ and for which the quantum efficiency of ferrioxalate decomposition has been established (*Φ* = 1.01). We conducted the quantum yield measurements in a standard fluorescence spectrophotometer capable of variable wavelength emission. First, in order to determine the intensity of the fluorometer at *λ* = 436 nm, we irradiated a solution containing a known concentration of ferrioxalate and quantified the appearance of Fe(ii) by UV-vis absorbance spectroscopy. From these data and the reported quantum yield of Fe(iii) reduction, we calculated a photon flux of 6.67 × 10^–10^ E s^–1^ from the fluorometer source.

**Scheme 3:**
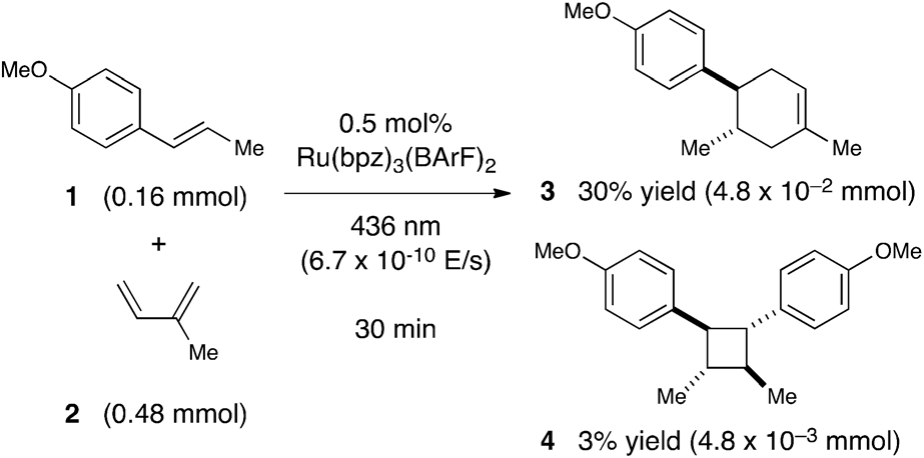
Calculating quantum yield for photocatalytic radical cation Diels–Alder cycloaddition.

**Fig. 1:**
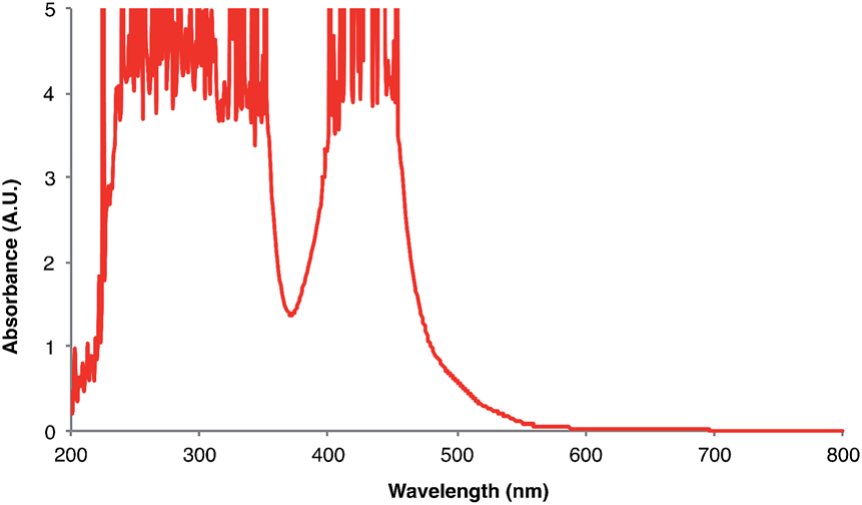
UV-vis absorption spectrum for Ru(bpz)_3_(BArF)_2_ at 4 × 10^–4^ M in CH_2_Cl_2_.

Next, we conducted a radical cation Diels–Alder cycloaddition in the fluorometer with 0.16 mmol anethole (**1**) and 0.48 mmol isoprene (**2**) in the presence of 0.5 mol% Ru(bpz)_3_^2+^ (Scheme 3). Importantly, despite the relatively low concentration of the photocatalyst (4.0 × 10^–4^ M), the optical transmittance at 436 nm was negligible (Fig. 1). Each of the organic coupling partners, on the other hand, was transparent at 436 nm, so we could make the limiting assumption that the incident photon flux is completely absorbed by the photocatalyst. After 30 min of irradiation in the fluorometer, we obtained 30% yield of Diels–Alder cycloadduct **3** (4.8 × 10^–2^ mmol). In addition, [2 + 2] homodimer **4**, a byproduct also arising from reaction of alkene radical cation **1˙^+^**,^24^ was formed in 3% yield (4.8 × 10^–3^ mmol). Thus, the overall quantum yield for formation of all radical cation cycloaddition products can be calculated by dividing the combined moles of **3** and **4** formed by the einsteins of photons consumed (eqn (1)); from these data, we calculate a quantum yield value of *Φ* = 44. In other words, 44 equivalents of product are formed for every photon absorbed by the photocatalyst, which is a result that could only be consistent with a chain mechanism.1
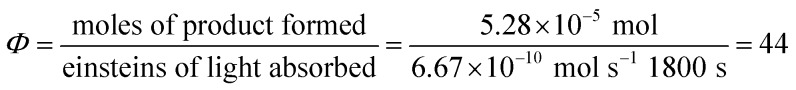

It is important to note that this calculation does not take into account the participation of any other photoinitiated processes that do not lead to product. For example, the photoexcited Ru*(bpz)_3_^2+^ catalyst could relax to the ground state *via* either radiative or vibrational pathways without undergoing electron-transfer processes; the reduced photocatalyst Ru(bpz)_3_^+^ and the oxidized alkene **1˙^+^** could also recombine to regenerate Ru(bpz)_3_^2+^ and neutral **1***via* back electron transfer. Crucially, any such non-productive processes would reduce the numerator of eqn (1) without affecting the denominator. Thus, although the observation of a quantum yield much greater than unity provides confirmation of the chain nature of this reaction, the actual length of the chains could be substantially higher than the quantum yield.

**Scheme 4:**
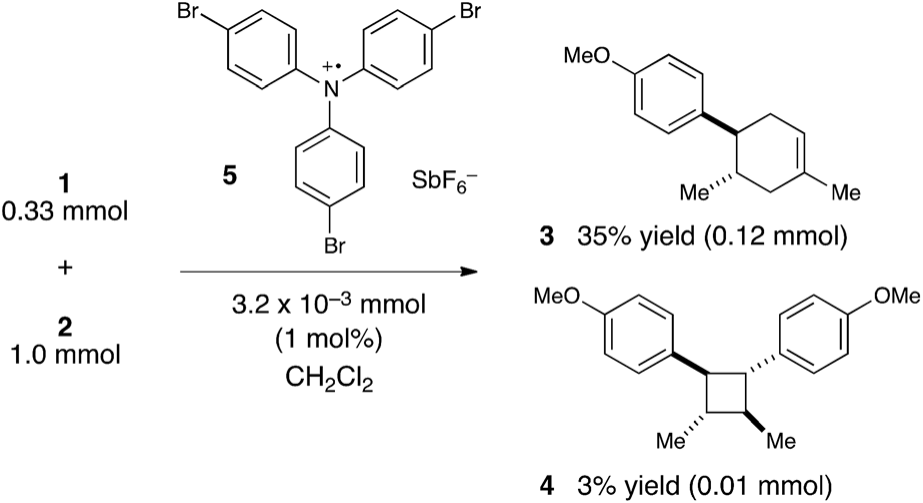
Calculating chain length for chemically induced radical cation Diels–Alder cycloaddition.

We were, however, intrigued by the observation that the quantum yield that we calculated is of the same order of magnitude as the chain lengths reported by Bauld for mechanistically similar [2 + 2] styrene radical cation cycloaddition reactions (*ca.* 20).^25^ This led us to wonder whether the quantum yield might indeed be a reasonable estimate for the length of the radical cation chains in the Diels–Alder cycloaddition. To enable a reasonable comparison, we chemically initiated a radical cation [4 + 2] cycloaddition of 0.33 mmol **1** and 1.0 mmol **2** using a catalytic quantity of triarylaminium cation **5** (3.2 × 10^–3^ mmol). In this experiment (Scheme 4), we observed the formation of 38% combined yield of **3** and **4** (0.13 mmol total). This corresponds to an average chain length of 41 (eqn (2)), a value within experimental error of the quantum yield that we calculated using eqn (1). Thus, in addition to providing compelling evidence that the photocatalytic radical cation Diels–Alder cycloaddition is a chain process, this study also suggested to us that quantum yield measurements might provide a convenient method to quickly estimate the average chain length involved in photocatalytic reactions. This conjecture is further interrogated in the next section.2
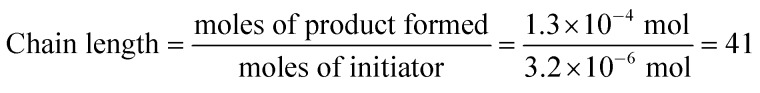

### Photoinitiated radical anion reactions

Much of the recent research in photoredox catalysis has been motivated by the fact that both oxidative and reductive one-electron transfer processes are readily accessible. The versatile redox properties of Ru(bpy)_3_^2+^ and similar photoredox catalysts provide uniquely direct access to a wide range of odd-electron reactive intermediates with diverse chemical behavior.^26^ In order to probe our hypothesis that chain mechanisms could be a general feature of photoredox reactions, we next elected to study the photocatalytic [2 + 2] cycloaddition of enones reported by our group several years ago.^27^ We selected this reaction as an example of a photoreductively initiated process that would contrast with the photooxidative radical cation Diels–Alder reaction described in the previous section. We hoped that evidence that both of these classes of reactions possess quantum yields greater than unity would provide further evidence supporting our contention that chain mechanisms are more common in photoredox chemistry than is generally appreciated.

**Scheme 5:**
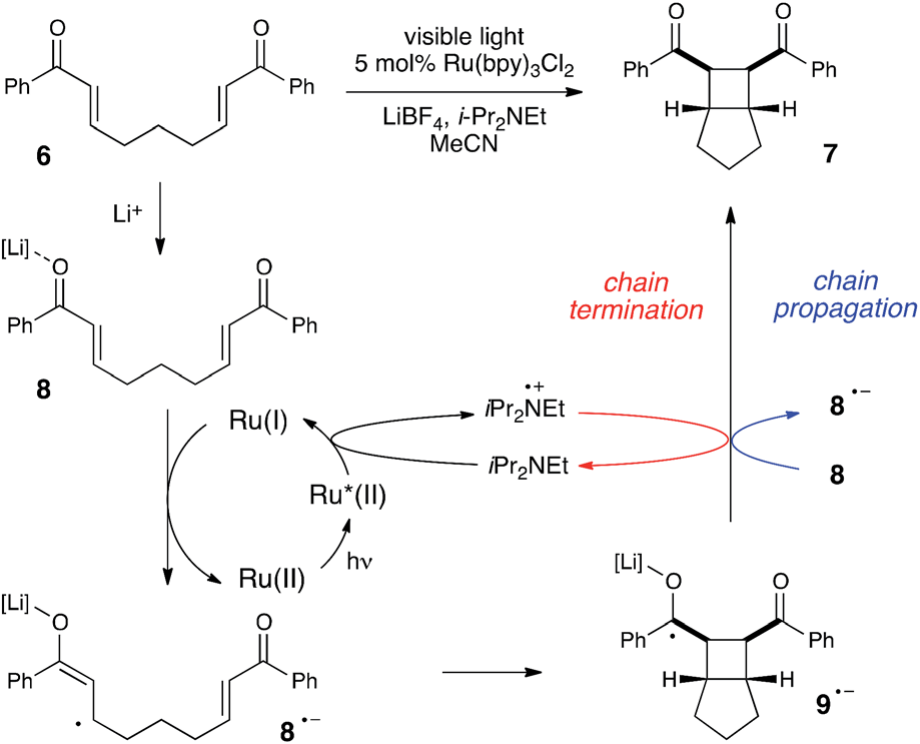
Mechanistic proposal for the radical anion [2 + 2] cycloaddition.

Scheme 5 depicts a working model for the mechanism of this transformation. We have proposed that the initiating step involves reductive quenching of photoexcited Ru*(bpy)_3_^2+^ by i-Pr_2_NEt. This step produces Ru(bpy)_3_^+^, which reacts with Lewis acid-activated enone **8** in a one-electron reduction process to generate the key radical anion **8˙^–^**. The enone radical anion then undergoes [2 + 2] cycloaddition to afford the cyclobutyl ketyl radical **9˙^–^**, which must lose an electron in order to generate the neutral product **7**. This final product-forming electron-transfer step could either be chain-terminating reduction of photogenerated amine radical cation i-Pr_2_NEt˙^+^, or chain-propagating reduction of another equivalent of Li-activated enone **8**.

Using the same experimental setup described in the previous section, we calculated a quantum yield of *Φ* = 77, again demonstrating that product formation is dominated by a chain process. However, we suspected that the average chain length might actually be somewhat longer than the quantum yield suggests on its own. In the course of measuring the quantum yield for this reaction, we observed that the reaction sample continued to luminesce visibly throughout the reaction. Thus, some non-negligible proportion of the photons absorbed by Ru(bpy)_3_^2+^ are re-emitted *via* phosphorescence, and only a fraction of the excited Ru*(bpy)_3_^2+^ triplets participate in productive electron transfer processes. This contrasts sharply to the radical cation Diels–Alder reaction, which displayed negligible luminescence compared to a blank sample of catalyst.^28^

**Fig. 2:**
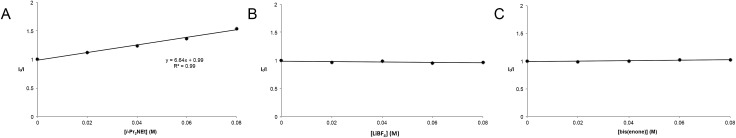
Stern–Volmer quenching studies for (A) i-Pr_2_NEt, (B) LiBF_4_, and (C) enone **6**.

The partitioning of excited state Ru*(bpy)_3_^2+^ between reductive quenching by i-Pr_2_NEt, which initiates the product-forming radical anion cycle, and non-productive relaxation pathways such as phosphorescence can be expressed as a quenching fraction (*Q*). The initial value of *Q* can be calculated using eqn (3), which expresses the quenching fraction as a ratio of the rate at which the excited photocatalyst is productively quenched by i-Pr_2_NEt to the sum of the rates of all of the relaxation processes available to the excited state. The intrinsic rate of all unimolecular radiative and non-radiative relaxation reactions of Ru*(bpy)_3_^2+^ is given by inverse of the lifetime of an excited state (*τ*_0_), a known quantity with a value of 855 ns in MeCN.^29^ The quenching rates (*k*_q_) for each of the reaction components can be directly measured using standard Stern–Volmer analyses, the results of which are shown in Fig. 2. Of the various reaction components, only i-Pr_2_NEt resulted in any measurable Stern–Volmer quenching (*k*_q,HB_ = 7.9 × 10^6^ M^–1^ s^–1^);^30^ we observed no change in Ru*(bpy)_3_^2+^ luminescence upon varying the concentrations of either LiBF_4_ or the enone substrate, consistent with the mechanism shown in Scheme 5. From these data, we calculated a quenching fraction of *Q* = 0.57.3
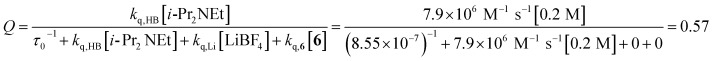



Thus the product-forming electron-transfer event is relatively inefficient: only 57% of the photons absorbed by the photocatalyst result in product-forming electron transfer, and 43% of the excited metal complexes relax *via* energy-wasting luminescence or internal conversion processes. The chain length, therefore, is more accurately approximated by dividing the calculated quantum yield by the quenching fraction. This analysis suggests that the average chain length of *Φ*/*Q* = 135 for the radical anion mediated intramolecular [2 + 2] cycloaddition of **6**.

**Fig. 3:**
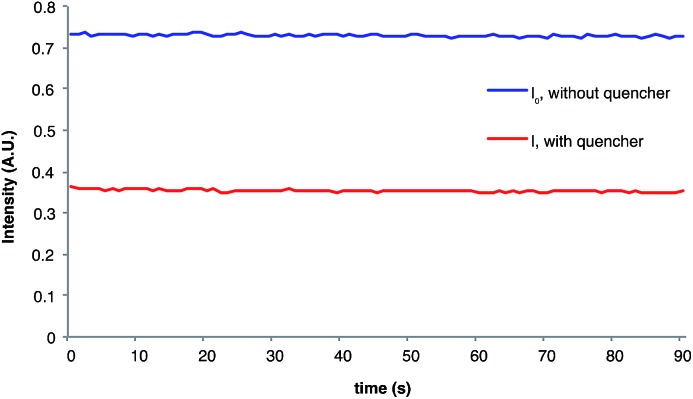
Phosphorescence intensity during reaction.

The measurement of Stern–Volmer quenching rates is a well-validated but somewhat time-consuming process. For operational simplicity, we wondered if a more rapid estimation of the initial quenching fraction might be available by comparing the phosphorescence of the reaction in progress to a control sample of the unquenched photocatalyst. This experiment is facilitated by the fact that we determined quantum yields using an irradiation source capable of simultaneous luminescence detection. Indeed, the phosphorescence intensity (*I*) of the catalyst under radical anion [2 + 2] cycloaddition reaction conditions is 50% that of the catalyst when i-PrNEt_2_ is omitted (*I*_0_), which provides an estimated initial quenching fraction within experimental error of the value calculated from a complete Stern–Volmer analysis (Fig. 3). The chain length derived from this value is calculated by dividing the measured total quantum yield by the fraction of catalysts whose phosphorescence is quenched (1 – *I*/*I*_0_), which gives a calculated value for average chain length of 154.^31^,^32^

### Photoinitiated reactions of neutral radicals

Photoredox processes result in the formation of radical ion intermediates that can be induced to directly participate in a variety of productive transformations such as those described in the previous two sections. A very common alternative mode of reactivity in photoredox catalysis involves a secondary fragmentation of photogenerated radical ions into discrete radical and ionic species, thereby affording access into the rich chemistry of neutral radical intermediates.

**Scheme 6:**
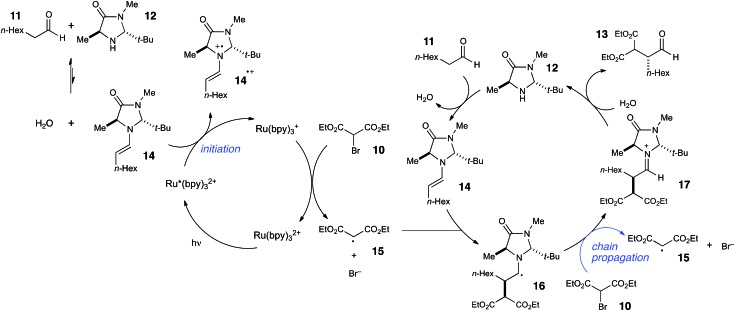
Mechanistic proposal for organocatalytic photoredox α-alkylation of aldehydes.

An important, seminal example of this reactivity is the asymmetric α-alkylation reaction reported by MacMillan (Scheme 6).^33^ Mechanistically, this reaction involves two interacting catalytic cycles and is thus somewhat more complicated than the prior two examples. Nevertheless, it shares several similar essential features. First, the reaction is proposed to be initiated by reductive quenching of Ru*(bpy)_3_^2+^ by a sacrificial quantity of enamine **14**, generated by condensation of organocatalyst **12** with aldehyde substrate **11**. Second, the ultimate, closed-shell product **13** arises from one-electron oxidation of **16** followed by hydrolysis of the resulting iminium. MacMillan has proposed a closed catalytic cycle in which this final oxidation is a chain-terminating electron-transfer to Ru*(bpy)_3_^2+^. We propose that a more likely product-forming step would be chain-propagating reduction of the bromomalonate **10** by α-amino radical **16**, which would also be a quite exergonic process.

**Scheme 7:**
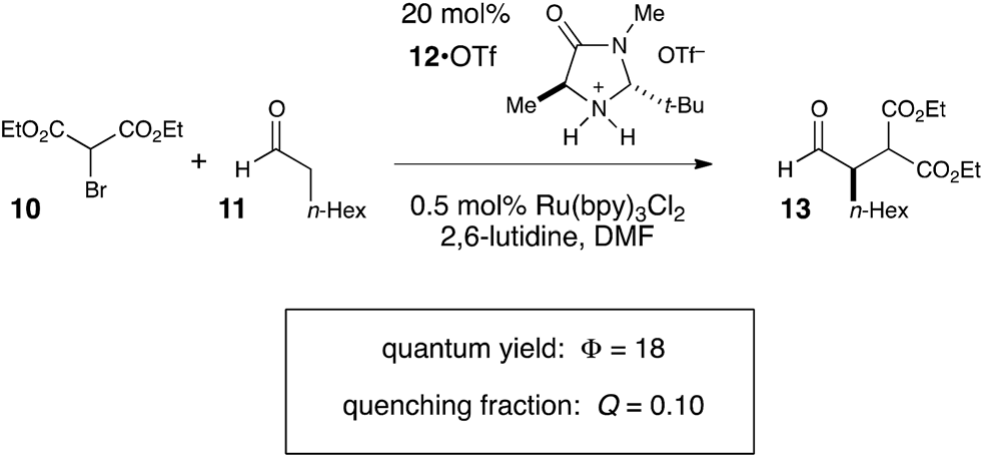
Quantum yield measurement for the organocatalytic photoredox α-aldehyde alkylation.

We studied the reaction of **10** with **11** (Scheme 7) and found that this reaction possesses a quantum yield of *Φ* = 18, again signifying a chain mechanism. Surprisingly, this value is quite a bit larger than that determined by König and Riedle, who reported *Φ* = 0.49 for the same transformation.^34^ These two experiments involve experimental setups that differ in several ways that might account for the discrepancy between the two measurements, the most significant of which is the presence of oxygen, which we excluded in our experiments. Triplet dioxygen is a rapid and efficient quencher of Ru*(bpy)_3_^2+^;^35^ by sparging the reaction solution with N_2_, we eliminate a major source of inefficiency in the photocatalytic α-alkylation reaction that would otherwise negatively impact the observed quantum yield. Indeed, our attempt to measure *Φ* under aerobic conditions using our experimental setup were unsuccessful because the low intensity of the spectrophotometer source resulted in only trace conversion after 4 h of irradiation.

This reemphasizes the important point that many non-productive processes, including parasitic quenching by energy or electron transfer as well as unimolecular decay processes, can result in a loss of measured quantum efficiency for a photochemical reaction. Indeed, in determining the quantum yield for the α-alkylation, we observed that the phosphorescence of the catalyst is only diminished by 10% under our experimental conditions. Thus, only a small fraction of the photoexcited Ru*(bpy)_3_^2+^ complexes productively initiate product-forming chains before they relax to the ground state by non-productive phosphorescence or internal conversion. This observation indicates that the radical chain lengths are in fact quite long, with a lower limit of 180.

The combination of quantum yield and luminescence quenching measurements, therefore, reveals that the α-alkylation reaction involves long radical chains but a relatively inefficient initiation process. We can rationalize the poor phosphorescence quenching as a consequence of several factors. First, the equilibrium for formation of enamine **14** is unfavorable; we measured *K*_eq_ = 8.1 × 10^–3^. Second, organocatalyst **12** is used at sub-stoichiometric loadings, resulting in a quite low concentration of initiating enamine. Finally, MacMillan reports a small Stern–Volmer quenching constant of 10 M^–1^ for **14**.^33^ These observations provide a satisfying explanation for the inefficient rate of initiation and for the relatively high reaction concentration required for successful reaction (0.5 M in **10**).

**Fig. 4:**
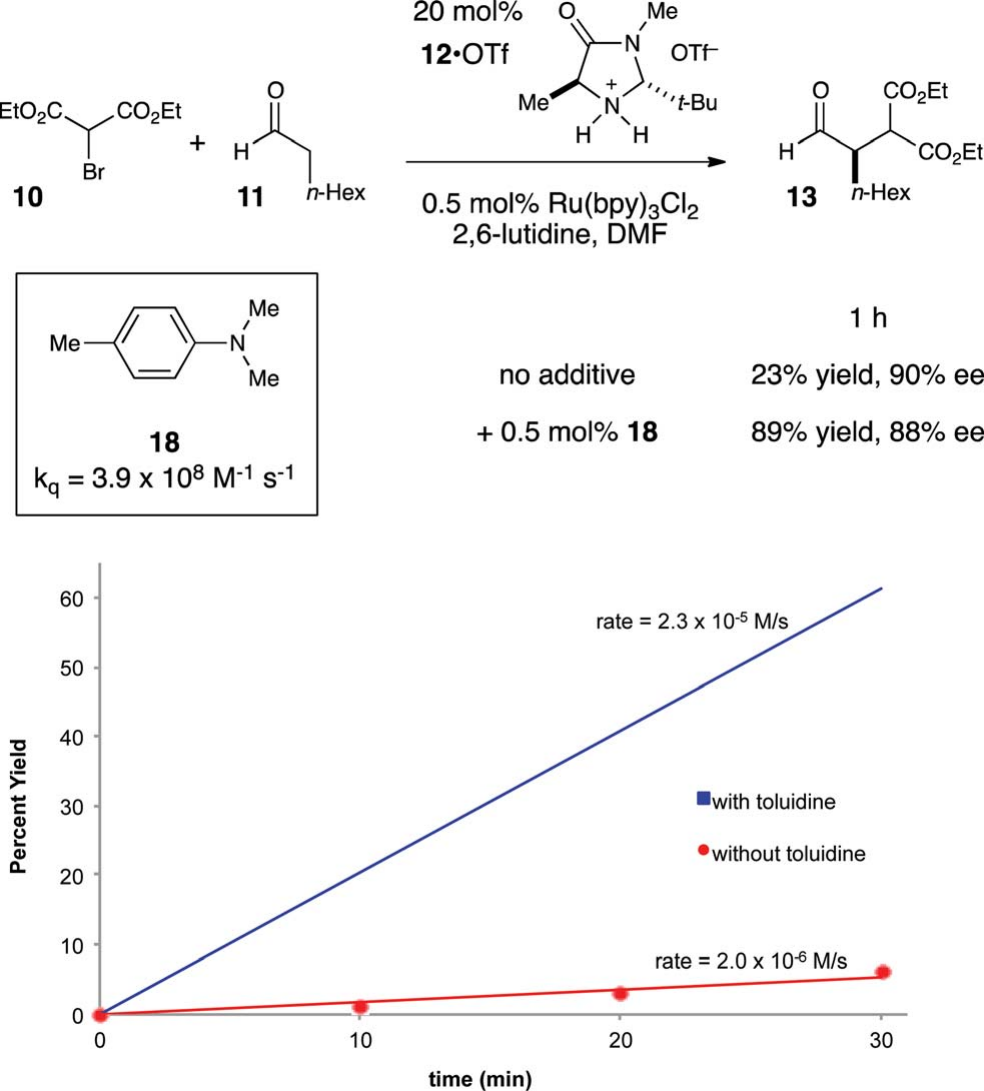
Effect of exogenous co-catalytic reductive quencher.

This understanding of the mechanism of the photoredox α-alkylation reaction suggested to us that a simple, rational modification might dramatically improve its rate. If our model for the mechanism of this reaction is correct, then the addition of a catalytic quantity of a co-reductant that could reductively quench Ru*(bpy)_3_^2+^ at faster rates than enamine **14** would be expected to increase the efficiency of chain initiation and thus improve the overall rate of product formation. To probe this hypothesis, we elected to study the effect of *N*,*N*-dimethyl-*p*-toluidine (**18**) on this reaction (Fig. 4). We selected this additive because it is known to quench Ru*(bpy)_3_^2+^ at a very fast rate.^36^

Indeed, upon addition of only 0.5 mol% **18**, we observed that the rate of product formation increased by over an order of magnitude (Fig. 4). When the reaction was allowed to proceed to a 1 h timepoint, the experiment conducted without **18** had progressed to only 23% yield; the reaction conducted with co-catalytic **18**, on the other hand, was complete (89% yield), and the product was formed with essentially the same enantioselectivity in both cases. Control experiments conducted in the absence of Ru(bpy)_3_Cl_2_ showed that **18** had no impact on the rate of the direct photoreaction.^37^ Thus, we conclude that **18** improves the rate of the reaction by accelerating an otherwise inefficient radical chain initiation step, consistent with our guiding hypothesis.

Thus, we would suggest that these experiments are valuable for a variety of reasons. First, the ability to rapidly determine chain lengths in photocatalytic reactions can provide valuable information on whether product formation is dominated by single-turnover catalyst-mediated steps or by catalyst-free chain propagation reactions, a detail that is important for fully understanding the mechanism of a photocatalytic reaction. Moreover, the ability to easily diagnose whether inefficiencies in a photoredox reaction are a result of short chain lengths or of slow initiation steps can provide valuable insights that can guide the rational optimization of the method. We suggest that this approach to studying the mechanism of photocatalytic reactions should be generally applicable to the growing body of literature involving photoredox catalysis.

### “Light/dark” experiments

**Fig. 5:**
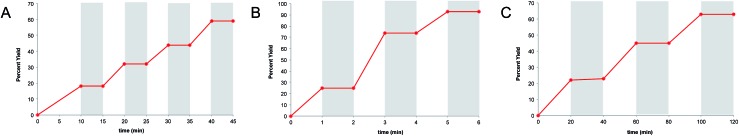
“Light/dark” experiments for (A) the radical cation [4 + 2] cycloaddition, (B) radical anion [2 + 2] cycloaddition, and (C) photoredox aldehyde alkylation reaction.

Finally, we wished to test whether “light/dark” experiments could in fact be used to unambiguously disprove the occurrence of chain processes in photoredox reactions. As described in the previous sections, we have obtained compelling evidence that the radical cation [4 + 2], radical anion [2 + 2], and neutral radical α-alkylation reactions all involve long product-forming chains. Nevertheless, when each of these reactions is conducted using alternating intervals of light and dark, we observe that product formation occurs only during periods of constant irradiation (Fig. 5a–c), similar to the results of other “light/dark” experiments recently reported in the literature.^12^

There are indeed a number of useful conclusions that can be drawn from “light/dark” experiments such as these. For instance, the observation that product is formed only upon constant irradiation suggests that a photocatalytic reaction might be susceptible to temporal and spatial control, a characteristic that can have important ramifications in materials applications.^4^,^38^ This behavior, however, can evidently still manifest in photocatalytic reactions involving long chain reactions, and we urge caution in drawing conclusions about chain propagation from “light/dark” experiments.

## Conclusions

We have demonstrated that chain processes dominate product formation in three representative photoredox transformations. While the conclusions drawn from these studies do not necessarily suggest that chain reactions occur in all photoredox reactions, the studies described in this paper do suggest that radical chain processes should be considered as a possibility when proposing the mechanisms of photoredox reactions, and that “light/dark” experiments cannot be used to conclusively rule them out. Further, we demonstrated that the combination of quantum yield measurements and luminescence quenching experiments provides a convenient method to rapidly determine a lower limit for chain lengths and to diagnose inefficient initiation steps in photoredox reactions. Our hope is that this simple approach to characterizing chain processes in photoredox reactions will become a routine analytical tool that will help to elucidate the fundamental mechanistic characteristics of this growing class of synthetically powerful transformations.

## Supplementary Material

Supplementary information
