# Correction: Characterizing chain processes in visible light photoredox catalysis

**DOI:** 10.1039/c5sc90050f

**Published:** 2015-08-28

**Authors:** Megan A. Cismesia, Tehshik P. Yoon

**Affiliations:** a Department of Chemistry , University of Wisconsin-Madison , 1101 University Avenue , Madison , Wisconsin 53706 , USA . Email: tyoon@chem.wisc.edu

## Abstract

Correction for ‘Characterizing chain processes in visible light photoredox catalysis’ by Megan A. Cismesia *et al.*, *Chem. Sci.*, 2015, DOI: 10.1039/c5sc02185e.



## 

Due to a production error, several of the data points in Fig. 4 were not displayed. The corrected version of Fig. 4 is shown below.

**Fig. 4:**
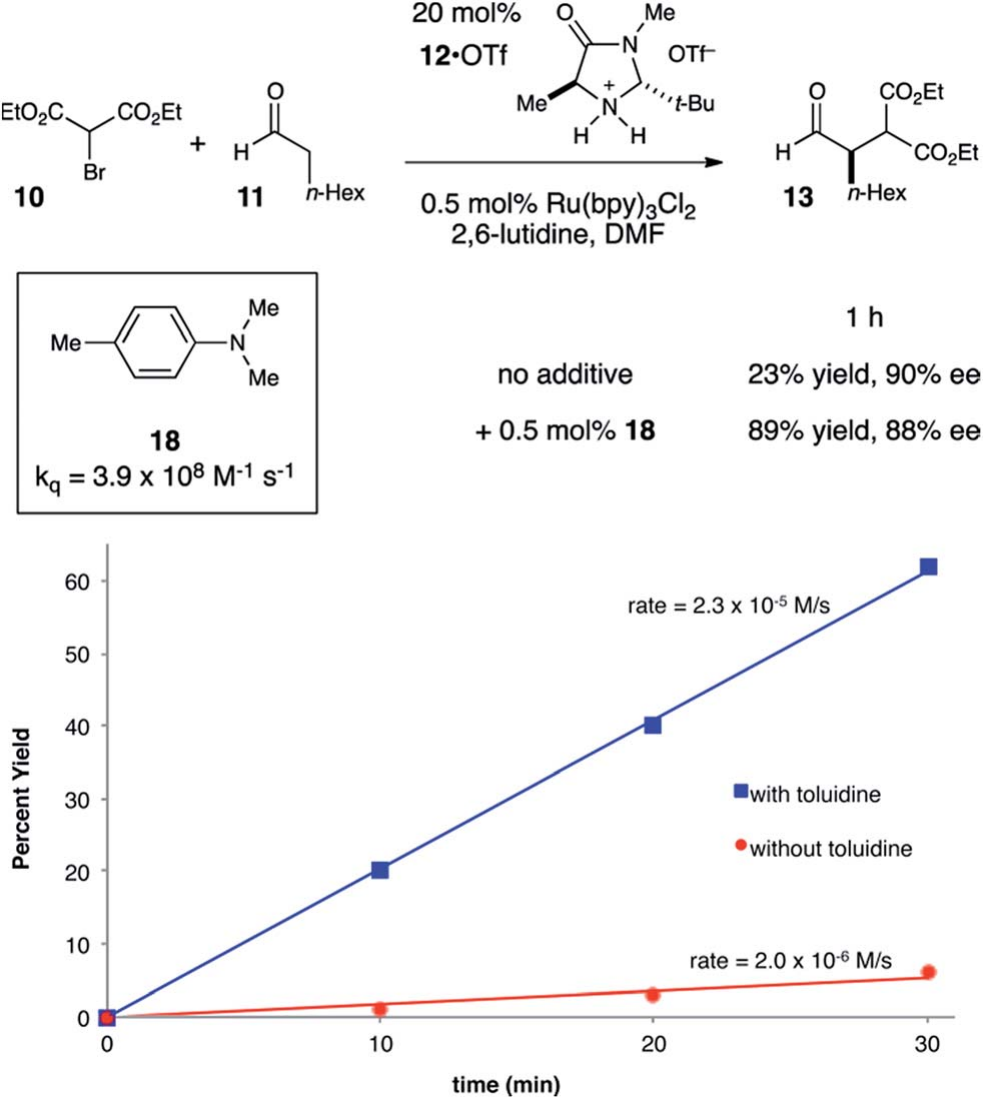
Effect of exogenous co-catalytic reductive quencher.

The Royal Society of Chemistry apologises for these errors and any consequent inconvenience to authors and readers.

