# Supplementary material for: Characterizing chain processes in visible light photoredox catalysis
Source: Chem Sci. 2015 Jul 7;6(10):5426–34. doi: 10.1039/c5sc02185e (PMC4676763; doi:10.1039/c5sc02185e)
Supplement: Supplementary file 1 [file SC-006-C5SC02185E-s001.pdf]

Electronic Supplementary Material (ESI) for Chemical Science.
This journal is © The Royal Society of Chemistry 2015

Supporting Information for:

Characterizing Chain Processes in Visible Light Photoredox Catalysis

Megan A. Cismesia and Tehshik P. Yoon*

Department of Chemistry, University of Wisconsin–Madison, 1101 University Avenue, Madison, Wisconsin 53706

I. General information S–1

II. Determination of light intensity S–2

III. [4+2] Reaction S–4

IV. [2+2] Reaction S–12

V. α-Alkylation reaction S–17

VI. *N*,*N*-Dimethyl-*p*-toluidine studies S–23

VII. References S–28

I. General information

Dichloromethane (CH_2Cl_2) and acetonitrile (MeCN) were purified through alumina as described by Grubbs.[1] Ru(bpz)$_3$(BArF)$_2$ was synthesized as previously reported.[2] Ru(bpy)$_3$Cl$_2$•6H$_2$O was purchased from Strem and used without further purification. Anethole, octanal, diethyl bromomalonate, and *N*,*N*-dimethyl-*p*-toluidine were purified *via* silica gel chromatography. Isoprene was distilled prior to use. Diisopropyl ethyl amine (*i*-Pr$_2$NEt) and 2,6-lutidine were distilled over calcium hydride, and *N*,*N*-dimethylformamide (DMF) was distilled over magnesium sulfate. Lithium tetrafluoroborate ($LiBF_4$) was purchased from Sigma–Aldrich and stored in a glovebox under an atmosphere of nitrogen. (*E*,*E*)-1,7-Dibenzoyl-1,6-heptadiene was prepared as previously reported (**6**)[3] and purified by flash chromatography immediately prior to use. (2*R*,5*S*)-2-*t*-Butyl-3,5-dimethylimidazolidine-4-one (**12**) was prepared as previously reported.[4] All solutions were prepared in the dark. Reactions were conducted in a 1 cm square quartz cuvette and capped with either a PTFE stopper or sealed with a rubber septum unless otherwise noted. A Hitachi F-4500 fluorescence spectrophotometer with a 150 W Xe lamp was used as the light source for the quantum yield measurements and luminescence quenching data. A 20 W compact fluorescent light bulb was used for "light/dark" and time course experiments at a distance of 8–10 cm away from the reaction flask. UV-vis data were measured on a Varian Cary 50 spectrophotometer. NMR data were measured on a Bruker AC 300 MHz or Bruker Avance 400 or 500 MHz spectrometer. The NMR facilites at UW–Madison are supported by the NSF (CHE-1048642, CHE-9208463) and NIH (S10 RR08389-01).

II. Determination of light intensity

Determination of the light intensity at 436 nm:

The photon flux of the spectrophotometer was determined by standard ferrioxalate actinometry.[5,6] A 0.15 M solution of ferrioxalate was prepared by dissolving 2.21 g of potassium ferrioxalate hydrate in 30 mL of 0.05 M H_2SO_4. A buffered solution of phenanthroline was prepared by dissolving 50 mg of phenanthroline and 11.25 g of sodium acetate in 50 mL of 0.5 M H_2SO_4. Both solutions were stored in the dark. To determine the photon flux of the spectrophotometer, 2.0 mL of the ferrioxalate solution was placed in a cuvette and irradiated for 90.0 seconds at λ = 436 nm with an emission slit width at 10.0 nm. After irradiation, 0.35 mL of the phenanthroline solution was added to the cuvette. The solution was then allowed to rest for 1 h to allow the ferrous ions to completely coordinate to the phenanthroline. The absorbance of the solution was measured at 510 nm. A non-irradiated sample was also prepared and the absorbance at 510 nm measured. Conversion was calculated using eq 1 .

$$\text{mol Fe}^{2+} = \frac{\text{V} \bullet \Delta\text{A}}{\text{l} \bullet \varepsilon} \tag{1}$$

Where V is the total volume (0.00235 L) of the solution after addition of phenanthroline, ΔA is the difference in absorbance at 510 nm between the irradiated and non-irradiated solutions, l is the path length (1.000 cm), and ε is the molar absorptivity at 510 nm (11,100 L mol^{-1} cm^{-1}).[5] The photon flux can be calculated using eq 2.

$$\text{photon flux} = \frac{\text{mol Fe}^{2+}}{\Phi \bullet \text{t} \bullet \text{f}} \tag{2}$$

Where Φ is the quantum yield for the ferrioxalate actinometer (1.01 for a 0.15 M solution at λ = 436 nm),[5] t is the time (90.0 s), and f is the fraction of light absorbed at λ = 436 nm (0.99833, *vide infra*). The photon flux was calculated (average of three experiments) to be 6.67×10^{-10} einstein s^{-1}.

Sample calculation:

$$\text{mol Fe}^{2+} = \frac{0.00235\ \text{L} \bullet 0.2868196}{1.000\ \text{cm} \bullet 11{,}100\ \text{L mol}^{-1}\ \text{cm}^{-1}} = 6.07 \times 10^{-8}\ \text{mol}$$

$$\text{photon flux} = \frac{6.07 \times 10^{-8}\ \text{mol}}{1.01 \bullet 90.0\ \text{s} \bullet 0.99833} = 6.69 \times 10^{-10}\ \text{einstein s}^{-1}$$

Determination of fraction of light absorbed at 436 nm for the ferrioxalate solution:

The absorbance of the above ferrioxalate solution at 436 nm was measured to be 2.777683. The fraction of light absorbed (f) by this solution was calculated using eq 3, where A is the measured absorbance at 436 nm.

$$f = 1 - 10^{-A} \qquad (3)$$

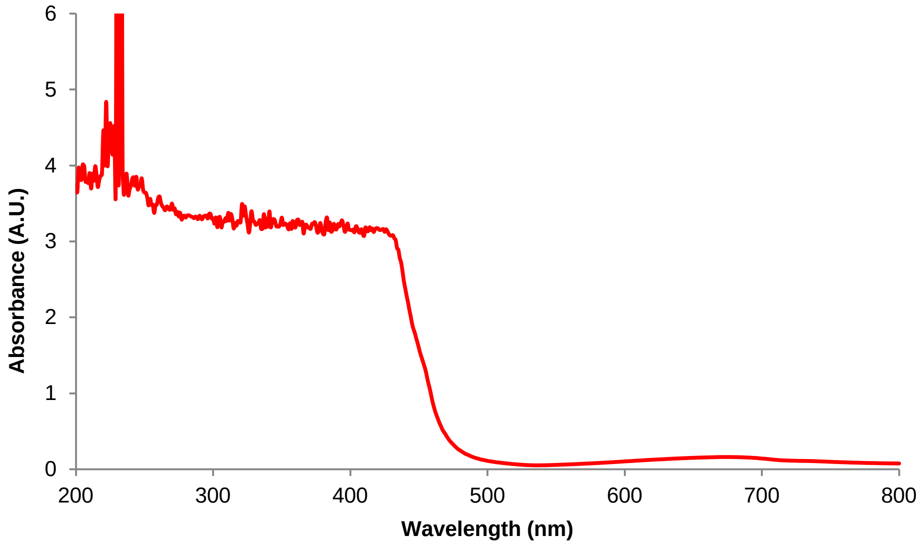

Figure 1. Absorbance of the ferrioxalate actinometer solution.

III. [4+2] Reaction[7]

Proposed mechanism:

Scheme 1: Proposed mechanism of the [4+2] radical cation Diels–Alder reaction between anethole (**1**) and isoprene (**2**).

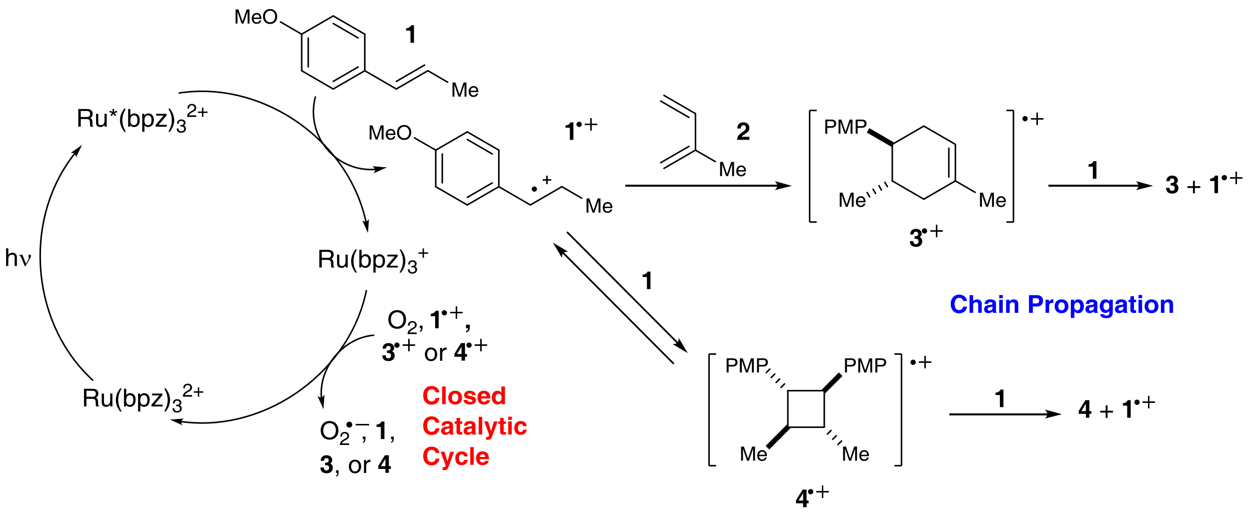

The reaction begins with $Ru(bpz)_3^{2+}$ absorbing a photon and generating the excited state. $Ru^*(bpz)_3^{2+}$ is quenched by **1**, forming both $Ru(bpz)_3^{+}$ and the radical cation $\mathbf{1}^{\bullet+}$. The reactivity of $\mathbf{1}^{\bullet+}$ can follow multiple pathways: (1) productive [4+2] formation with isoprene **2** to form $\mathbf{3}^{\bullet+}$, (2) reversible [2+2] cycloaddition with another molecule of **1** to generate $\mathbf{4}^{\bullet+}$, and (3) back electron transfer with $Ru(bpz)_3^{+}$ to regenerate **1** and the photocatalyst. In a closed catalytic cycle, $\mathbf{3}^{\bullet+}$ and $\mathbf{4}^{\bullet+}$ reoxidize $Ru(bpz)_3^{+}$ to regenerate the photocatalyst and form the neutral products. In a chain process, $\mathbf{3}^{\bullet+}$ and $\mathbf{4}^{\bullet+}$ oxidize a molecule of **1** to generate another equivalent of $\mathbf{1}^{\bullet+}$ and furnish the products.

Determination of quantum yield:

Scheme 2: [4+2] Reaction between anethole and isoprene.

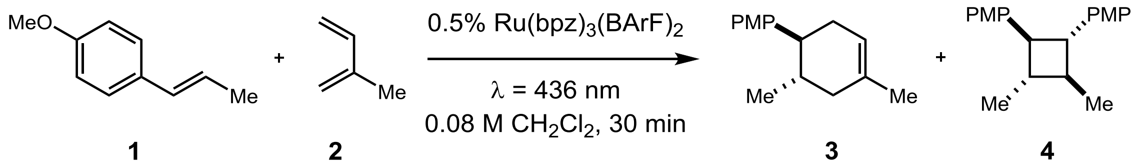

A cuvette was charged with anethole (0.16 mmol, 1 equiv), isoprene (0.48 mmol, 3 equiv), $Ru(bpz)_3(BArF)_2$ (0.00080 mmol, 0.5 mol%), and 2.0 mL CH_2Cl_2 (0.08 M). The cuvette was then capped with a PTFE stopper. The sample was stirred and irradiated (λ = 436 nm, slit width= 10.0 nm) for 1800 s (30 min). After irradiation, the solution was passed through a silica plug. The yield of product formed was determined by ^{1}H NMR based on a dibromomethane standard. The quantum yield was determined using eq 4. Essentially all incident light (f > 0.999, *vide infra*) is absorbed by the $Ru(bpz)_3(BArF)_2$ at the reaction conditions described above.

$$\Phi = \frac{\text{mol product}}{\text{flux} \bullet \text{t} \bullet \text{f}} \quad (4)$$

Experiment 1: 23.7 mg (0.16 mmol) anethole, 48 μL (0.48 mmol) isoprene, 1.8 mg (0.00078 mmol) $Ru(bpz)_3(BArF)_2$, 2.0 mL (0.08 M) CH_2Cl_2 after 1800 s yielded 30% of **3** and 3% of **4**. Φ(33%) = 44.

Sample quantum yield calculation:

$$\Phi = \frac{5.28 \times 10^{-5}\ \text{mol}}{6.67 \times 10^{-10} \text{einstein s}^{-1} \bullet 1800\ \text{s} \bullet 1.00} = 44$$

Experiment 2: 23.6 mg (0.16 mmol) anethole, 48 μL (0.48 mmol) isoprene, 1.8 mg (0.00078 mmol) $Ru(bpz)_3(BArF)_2$, 2.0 mL (0.08 M) CH_2Cl_2 after 1800 s yielded 31% of **3** and 2.5% of **4**. Φ(33.5%) = 44.

Absorbance of catalyst:

The absorbance of $Ru(bpz)_3(BArF)_2$ in CH_2Cl_2 was measured at the reaction concentration of 4.0 × 10^{-4} M and at a substantially more dilute concentration of 4.0 × 10^{-6} M. The absorbance at 436 nm for a 4.0 × 10^{-4} M solution is >3 indicating the fraction of light absorbed is >0.999.

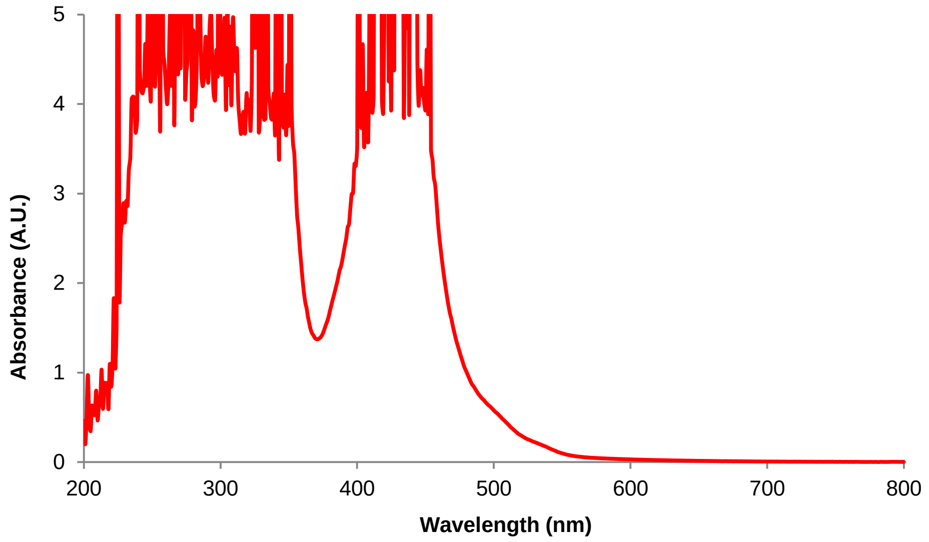

Figure 2. Absorbance of a 4.0 × 10^{-4} M solution of $Ru(bpz)_3(BArF)_2$ in CH_2Cl_2.

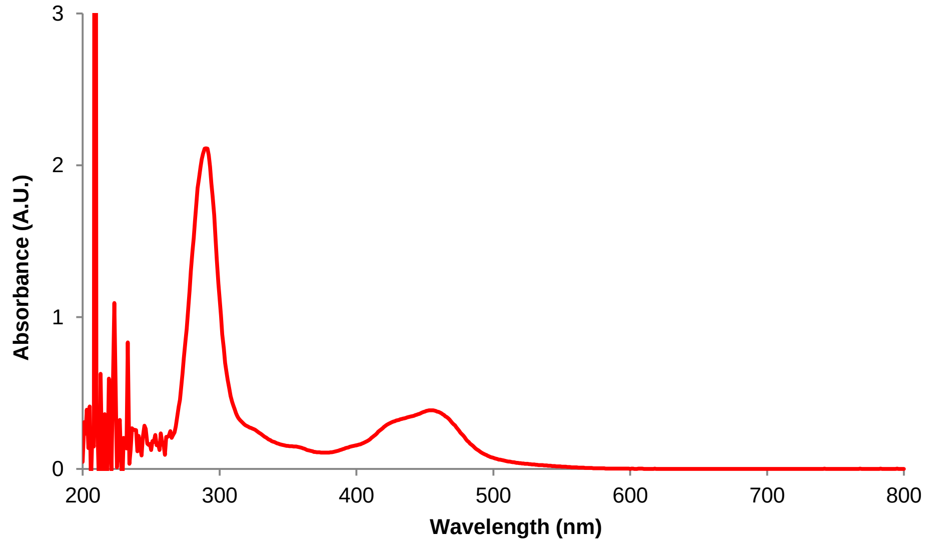

Figure 3. Absorbance of a 4.0 × 10^{-6} M solution of $Ru(bpz)_3(BArF)_2$ in CH_2Cl_2.

Lifetime measurement:

The excited state lifetime of $Ru(bpz)_3(BArF)_2$ in CH_2Cl_2 (395 ns, 3.9×10^{-5} M) was measured by the frequency-domain method with an ISS K2 spectrofluorometer. The excitation source was intensity modulated through varying MHz frequencies at the sample's absorption maximum, producing shifts in the intensity and phase of fluorescence emission. Comparison to a standard (in this case fluorescein and glycogen) allowed lifetime determination. Data was analyzed in Vinci (ISS).

Stern–Volmer quenching rate data:

Rates of quenching (k_q) were determined using Stern–Volmer kinetics (eq 5).

$$\frac{I_0}{I} = k_q \tau_0 [\text{quencher}] \tag{5}$$

Where I_0 is the luminescence intensity without the quencher, I is the intensity with the quencher, and τ_0 is the lifetime of the photocatalyst. For anethole and $Ru(bpz)_3(BArF)_2$, samples were prepared by adding solutions of photocatalyst, quencher, and CH_2Cl_2 to obtain a total volume of 2.0 mL. A glass cuvette with a 14/20 joint and screw cap was used. The cuvette was degassed by three freeze-pump-thaw cycles and then backfilled with N_2. The concentration of $Ru(bpz)_3(BArF)_2$ was 3.9×10^{-5} M. Samples were irradiated at 436 nm, and emission was detected at 558 nm.

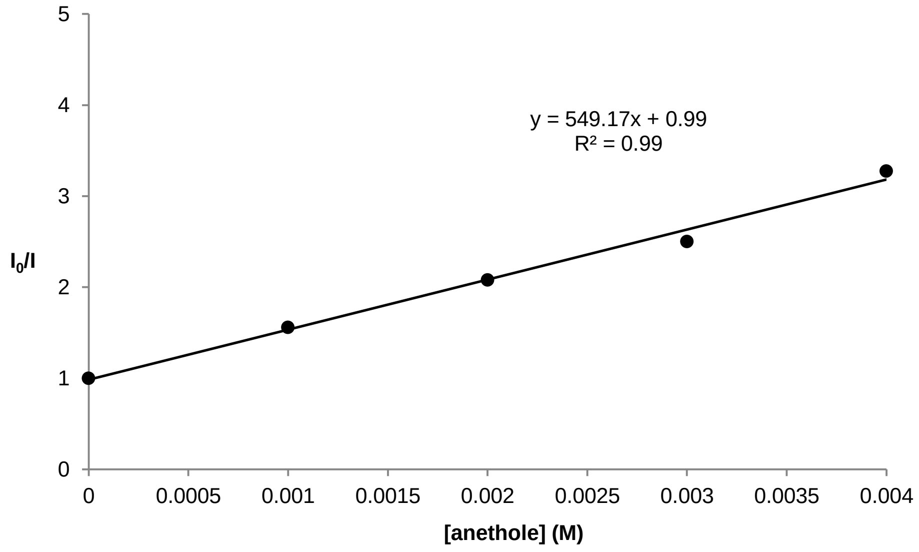

Figure 4. Stern–Volmer quenching experiment of $Ru(bpz)_3(BArF)_2$ and anethole. For anethole, $k_q = 1.4 \times 10^9\ M^{-1}\ s^{-1}$.

For isoprene and $Ru(bpz)_3(BArF)_2$, a cuvette containing a solution of catalyst in CH_2Cl_2 was degassed by three freeze-pump-thaw cycles and then backfilled with N_2. Samples were prepared by adding the solution of photocatalyst and isoprene (sparged) to a cuvette with a rubber septum under N_2. The concentration of $Ru(bpz)_3(BArF)_2$ was 3.9×10^{-5} M. The samples were irradiated at 436 nm, and emission was detected at 558 nm.

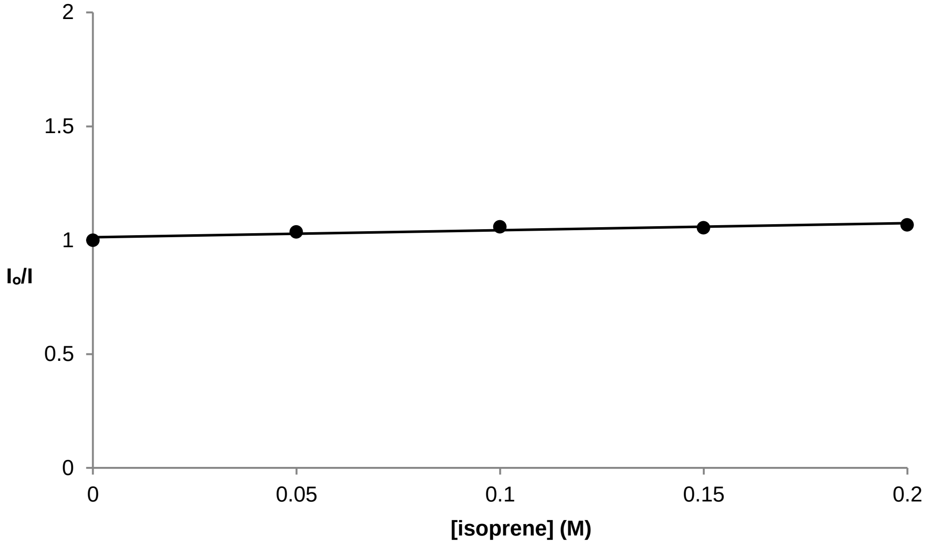

Figure 5. Stern–Volmer quenching experiment of $Ru(bpz)_3(BArF)_2$ and isoprene. No quenching observed.

For oxygen and $Ru(bpz)_3(BArF)_2$, values for k_q in MeCN have previously been reported.[8] The concentration of oxygen was calculated using Henry's Law.[9]

The quenching fraction, Q, was calculated using eq 6, where other processes include quenching through non-productive pathways (*e.g.* quenching by oxygen).

$$Q = \frac{k_q[\text{quencher}]}{\tau_0^{-1} + k_q[\text{quencher}] + \text{other processes}} \qquad (6)$$

Quenching fraction calculation:

$$Q = \frac{k_{q,\text{anethole}}[\text{anethole}]}{\tau_0^{-1} + k_{q,\text{anethole}}[\text{anethole}] + k_{q,O_2}[O_2]}$$

$$= \frac{1.4 \times 10^9\ M^{-1}s^{-1}[0.080\ M]}{\frac{1}{3.95 \times 10^{-7}\ s} + 1.4 \times 10^9\ M^{-1}s^{-1}[0.080\ M] + 2.6 \times 10^8\ M^{-1}s^{-1}[0.0019\ M]}$$

$$= 0.97$$

Simple quenching experiment:

The luminescence intensity under the reaction conditions (I) was recorded (λ = 558 nm, 10.0 nm slit width) while being irradiated in the fluorometer for the quantum yield measurement. Luminescence intensity without quencher (I_0) was also recorded for each reaction under the standard reaction conditions but excluding anethole. The quenching fraction, Q, was determined by eq 7 and averaged over the first 90 s of the reaction.

$$Q = \frac{I_0 - I}{I_0} \tag{7}$$

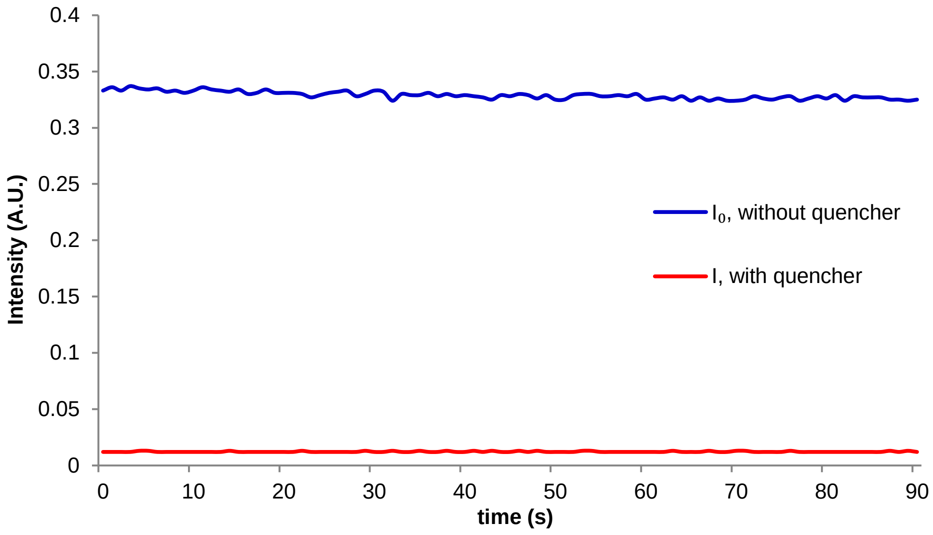

Figure 6. Phosphorescence intensity of the reaction over 90 s with (red line) and without (blue line) anethole.

For this reaction, Q = 0.96.

Chain length calculation:

Chain length values calculated in this paper are a lower limit approximation of the actual chain lengths and were calculated using eq 8, where Q was calculated either through the Stern–Volmer analysis or the simple quenching experiment.

$$\text{chain length} = \frac{\Phi}{Q} \tag{8}$$

Sample chain length calculation:

$$\text{chain length} = \frac{44}{0.97} = 45$$

For the Stern–Volmer analysis, the chain length was calculated to be 45; for the simple quenching experiment, the chain length was 46.

Light/dark experiment:

A vial was equipped with a stir bar and charged with 23.5 mg (0.16 mmol) anethole, 48 µL (0.48 mmol) isoprene, 1.7 mg (0.00074 mmol) $Ru(bpz)_3(BArF)_2$, 10.0 µL (0.058 mmol) trimethyl(phenyl)silane, and 2.0 mL (0.08 M) CD_2Cl_2. The reaction was stirred under ambient atmosphere. The reaction was alternatively irradiated with a 20 W CFL bulb and kept in the dark in five minute intervals. Aliquots were removed at the start and after each interval, passed through a silica plug, and diluted with $CDCl_3$. Yields of the **3** and **4** were determined by ^{1}H NMR and based on trimethyl(phenyl)silane as an internal standard.

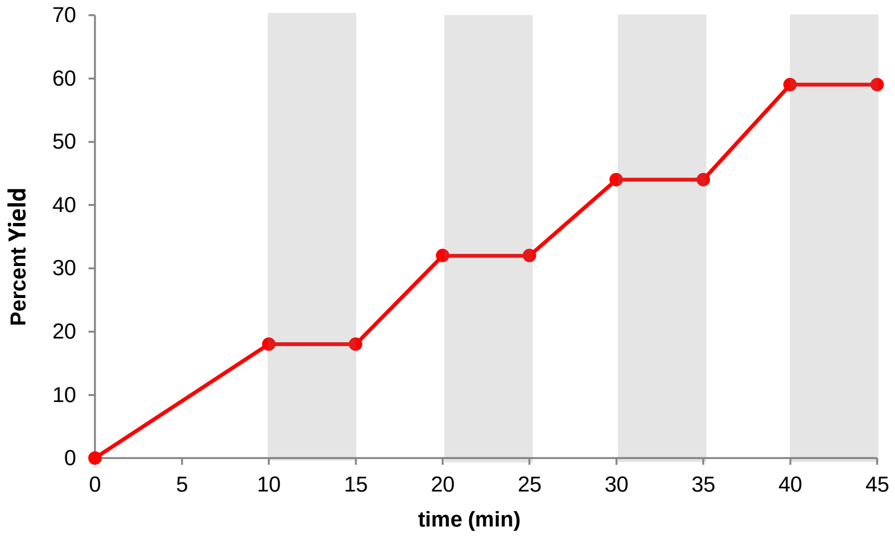

Figure 7. Light/dark experiment.

Non-photochemical [4+2] reaction:

Scheme 3. Aminium initiated [4+2] reaction between anethole and isoprene.

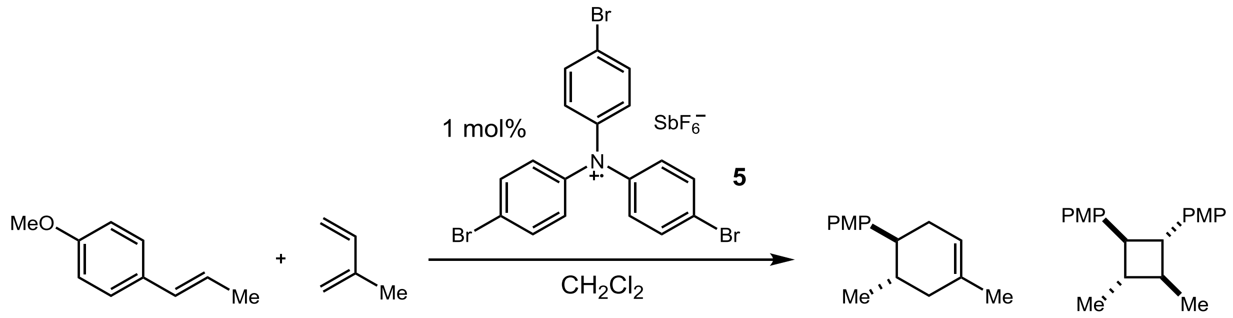

1 **2** **3** **4**

A vial was equipped with a stir bar and charged with 49.5 mg (0.33 mmol) anethole, 100 μL (1.0 mmol) isoprene, 2.6 mg (0.0032 mmol) tris(4-bromophenyl)ammoniumyl hexachloroantimonate (**5**), and 4.2 mL (0.08 M) CH_2Cl_2. The reaction was stirred for 1 h and then passed through a silica plug. The yield of product formed was determined by ^{1}H NMR based on trimethyl(phenyl)silane as an internal standard. The chain length was calculated by eq 9.

$$\text{chain length} = \frac{\text{mol } \mathbf{3} + \text{mol } \mathbf{4}}{\text{mol initiator}} \tag{9}$$

The above reaction yielded 0.12 mmol of **3** and 0.010 mmol of **4**. The chain length was calculated to be 41.

IV. [2+2] Reaction[10]

Determination of quantum yield:

Scheme 4. [2+2] cycloaddition of bis(enone) **6**.

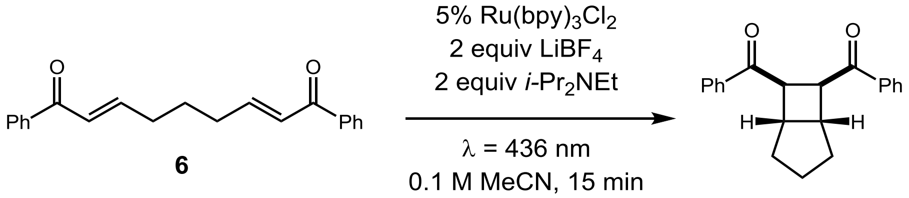

6 **7**

A cuvette was charged with bis(enone) **6** (0.20 mmol, 1 equiv), *i*-Pr_2NEt (0.40 mmol, 2 equiv), $LiBF_4$ (0.40 mmol, 2 equiv), $Ru(bpy)_3Cl_2•6H_2O$ (0.010 mmol, 5 mol%), and 2.0 mL MeCN (0.1 M). The cuvette was sealed with a rubber septum and parafilm, then degassed by sparging with N_2 for 15 min. The reaction was stirred and irradiated (λ = 436 nm, slit width = 10.0 nm) for 900 s (15 min). After irradiation, the solution was passed through a silica plug. The yield of product formed was determined by ^{1}H NMR based on a dibromomethane standard. The quantum yield was determined using eq 4. Essentially all incident light (f > 0.999, *vide infra*) is absorbed by the $Ru(bpy)_3Cl_2$ at the reaction conditions described above.

Experiment 1: 60.8 mg (0.20 mmol) of **6**, 70 μL (0.40 mmol) *i*-Pr_2NEt, 37.5 mg (0.40 mmol) $LiBF_4$, 7.5 mg $Ru(bpy)_3Cl_2•6H_2O$ (0.010 mmol), 2.0 mL MeCN after 900 s yielded 23% of **7**. Φ(23%) = 77.

Experiment 2: 60.7 mg (0.20 mmol) of **6**, 70 μL (0.40 mmol) *i*-Pr_2NEt, 37.5 mg (0.40 mmol) $LiBF_4$, 7.5 mg $Ru(bpy)_3Cl_2•6H_2O$ (0.010 mmol), 2.0 mL MeCN after 900 s yielded 23% of **7**. Φ(23%) = 76.

Absorbance of catalyst:

The absorbance of $Ru(bpy)_3Cl_2$ in MeCN at a concentration of 1.0×10^{-3} M (20% the reaction conditions above) was measured. Absorbance at 436 nm is >3 indicating the fraction of light absorbed is >0.999.

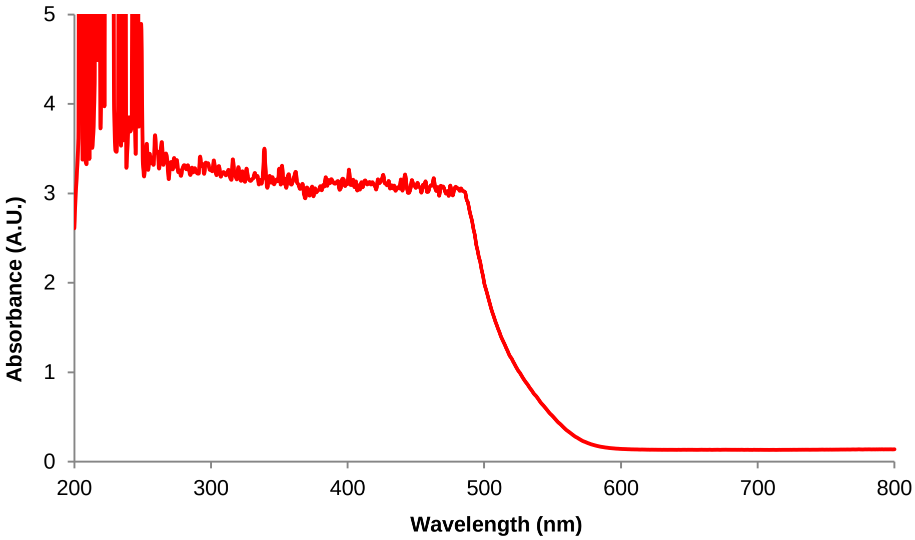

Figure 8. Absorbance of a 1.0×10^{-3} M solution of $Ru(bpy)_3Cl_2$ in MeCN.

Stern–Volmer quenching rate data:

Samples were prepared by adding solutions of photocatalyst, quencher, and MeCN to obtain a total volume of 2.0 mL. The cuvette was sealed with a septum and parafilm, and then sparged for 15 min with N_2. The concentration of $Ru(bpy)_3Cl_2$ was 5.0×10^{-5} M. Samples were irradiated at 451 nm, and emission was detected at 600 nm. The lifetime measurement for $Ru(bpy)_3Cl_2$ in MeCN (855 ns) was previously reported.[11]

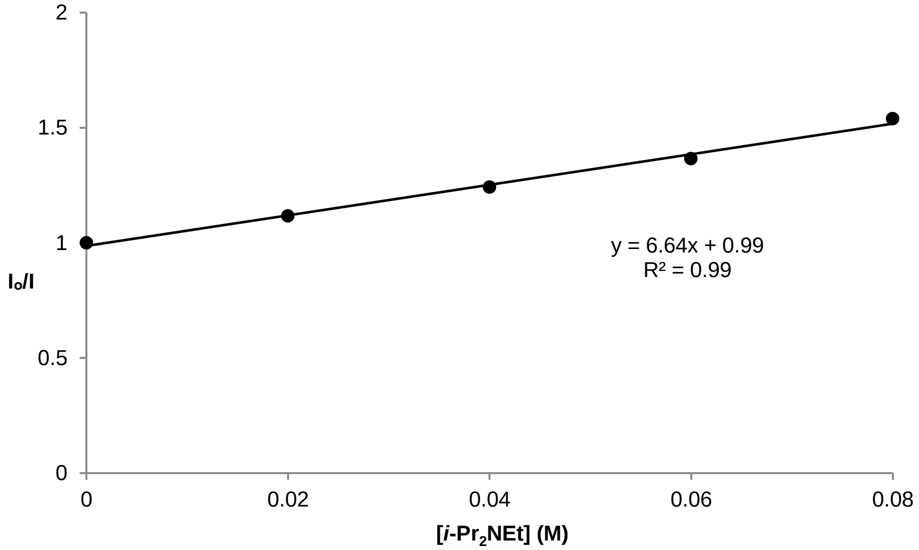

Figure 9. Stern–Volmer quenching of $Ru(bpy)_3Cl_2$ and *i*-Pr_2NEt. For the amine, $k_q = 7.8 \times 10^6$ M^{-1} s^{-1}.

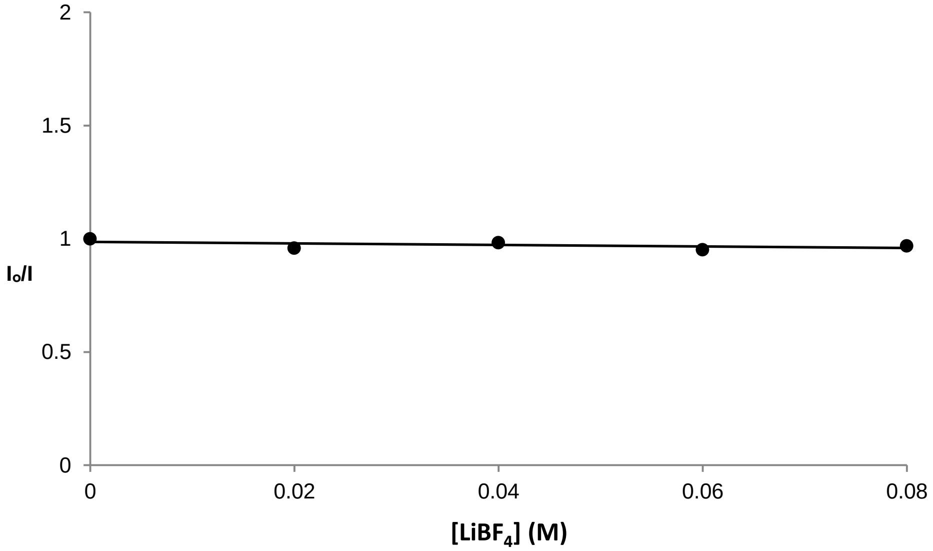

Figure 10. Stern–Volmer quenching of $Ru(bpy)_3Cl_2$ and $LiBF_4$. No quenching observed.

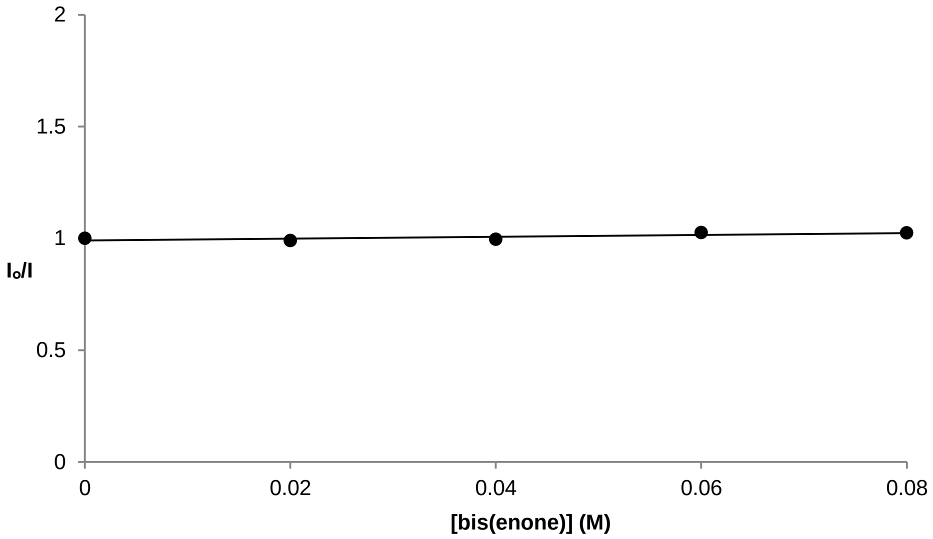

Figure 11. Stern–Volmer quenching of $Ru(bpy)_3Cl_2$ and the bis(enone) **6** starting material. No quenching observed.

Quenching fraction calculation:

$$Q = \frac{k_{q,i\text{-}Pr_2NEt}[i\text{-}Pr_2NEt]}{\tau_0^{-1} + k_{q,i\text{-}Pr_2NEt}[i\text{-}Pr_2NEt]}$$

$$= \frac{7.8 \times 10^6\ M^{-1}s^{-1}[0.20\ M]}{\frac{1}{8.55 \times 10^{-7}\ s} + 7.8 \times 10^6\ M^{-1}s^{-1}[0.20\ M]}$$

$$= 0.57$$

Simple quenching experiment:

The luminescence intensity under the reaction conditions (I) was recorded (λ = 600 nm, 10.0 nm slit width) while being irradiated in the fluorometer for the quantum yield measurement. Luminescence intensity without quencher (I_0) was also recorded for each reaction under the standard reaction conditions but excluding *i*-Pr_2NEt. The quenching fraction was determined by eq 7 and averaged over the first 90 s of the reaction.

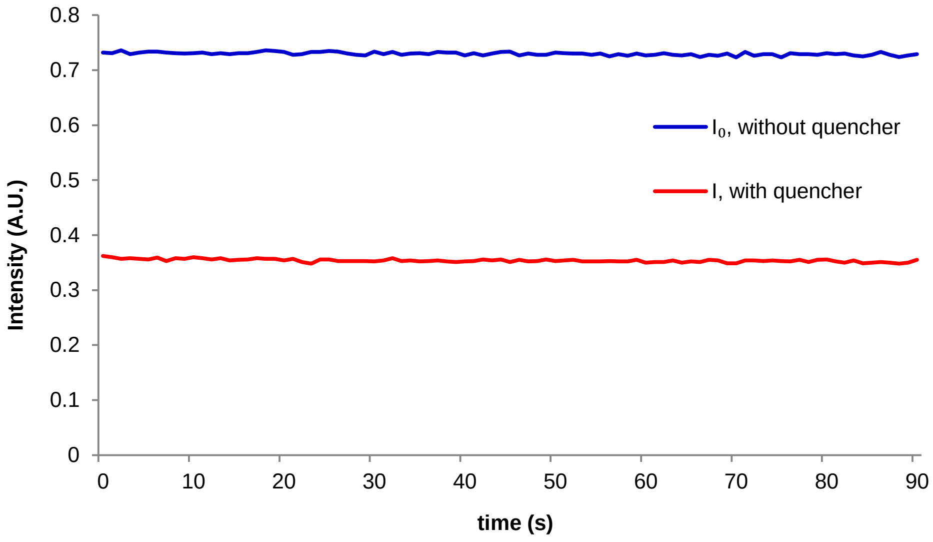

Figure 12. Phosphorescence intensity of the reaction over 90 s with (red line) and without (blue line) *i*-Pr_2NEt.

For this reaction, Q = 0.50.

Chain length calculation:

Chain length values calculated in this paper are a lower limit approximation of the actual chain lengths and were calculated using eq 8, where Q was calculated either through the Stern–Volmer analysis or the simple quenching experiment.

For the Stern–Volmer analysis, the chain length was calculated to be 135; for the simple quenching experiment, the chain length was 154.

Light/dark experiment:

A J. Young NMR tube was charged with 30.6 mg (0.10 mmol) **6**, 35 µL (0.20 mmol) *i*-Pr_2NEt, 18.8 mg (0.20 mmol) $LiBF_4$, 3.6 mg (0.048 mmol) $Ru(bpy)_3Cl_2•6H_2O$, 10.0 µL (0.058 mmol) trimethyl(phenyl)silane, and 1.0 mL (0.1 M) CD_3CN. The vessel was degassed by three freeze-pump-thaw cycles and backfilled with N_2. The reaction was alternatively irradiated with a 20 W CFL bulb and kept in the dark in one minute intervals until the reaction had reached completion. Yields of **7** at time points at the start and after each interval were determined by ^1H NMR and based on trimethyl(phenyl)silane as an internal standard.

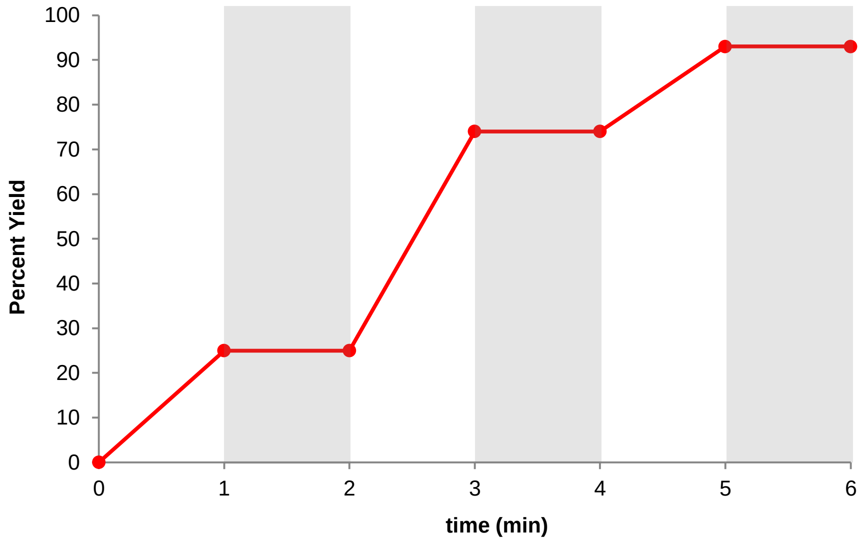

Figure 13. Light/dark experiment.

V. α-Alkylation reaction[12]

Determination of quantum yield (degassed):

Scheme 5. Asymmetric α-alkylation of octanal by diethyl bromomalonate.

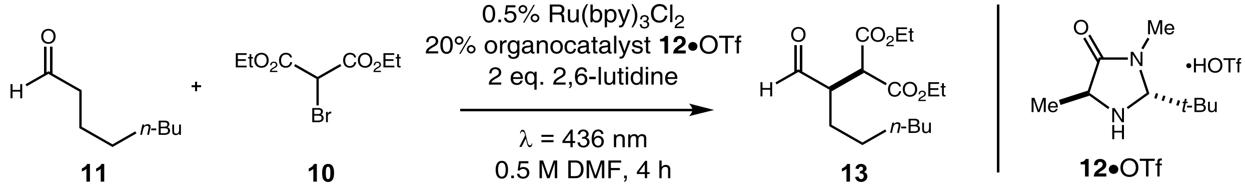

A cuvette was charged with octanal (1.3 mmol, 2 equiv), diethyl bromomalonate (0.65 mmol, 1 equiv), $Ru(bpy)_3Cl_2•6H_2O$ (0.0032 mmol, 0.5 mol%), the organocatalyst **12**•OTf (0.13 mmol, 20 mol%), 2,6-lutidine (1.3 mmol, 2 equiv) and 1.3 mL DMF (0.5 M). The cuvette was sealed with a rubber septum and parafilm. The cuvette was degassed by sparging with N_2 for 15 min. The reaction was stirred and irradiated (λ = 436 nm, slit width= 10.0 nm) for 14400 s (4 h). After irradiation, the solution was passed through a silica column. The yield of product formed was determined by ^{1}H NMR based on a trimethyl(phenyl)silane standard. The quantum yield was determined using eq 4. Essentially all incident light (f > 0.999, *vide infra*) is absorbed by the $Ru(bpy)_3Cl_2$ at the reaction conditions described above.

Experiment 1: 200 µL octanal (1.3 mmol), 110 µL diethyl bromomalonate (0.65 mmol), 2.4 mg $Ru(bpy)_3Cl_2•6H_2O$ (0.0032 mmol), 41.3 mg of **12**•OTf (0.13 mmol), 150 µL 2,6-lutidine (1.3 mmol) and 1.3 mL DMF after 14400 s yielded 27% of **13**. Φ(27%) = 18.

Experiment 2: 200 µL octanal (1.3 mmol), 110 µL diethyl bromomalonate (0.65 mmol), 2.4 mg $Ru(bpy)_3Cl_2•6H_2O$ (0.0032 mmol), 41.4 mg of **12**•OTf (0.13 mmol), 150 µL 2,6-lutidine (1.3 mmol) and 1.3 mL DMF after 14400 s yielded 27% of **13**. Φ(27%) = 18.

Determination of the quantum yield (under air):

A cuvette was charged with 200 µL octanal (1.3 mmol, 2 equiv), 110 µL diethyl bromomalonate (0.65 mmol, 1 equiv), 2.4 mg $Ru(bpy)_3Cl_2•6H_2O$ (0.0032 mmol, 0.5 mol%), 41.3 mg **12**•OTf (0.13 mmol, 20 mol%), 150 µL 2,6-lutidine (1.3 mmol, 2 equiv) and 1.3 mL DMF (0.5 M). The cuvette was capped with a PTFE stopper. The reaction was stirred and irradiated (λ = 436 nm, slit width= 10.0 nm) for 14400 s (4 h). After irradiation, the solution was passed through a silica column. Trace product formation (<1%) was observed in the ^{1}H NMR as compared to trimethyl(phenyl)silane standard.

Absorbance of catalyst:

The absorbance of $Ru(bpy)_3Cl_2$ in DMF was measured at the reaction concentration of 2.5×10^{-3} M. The absorbance at 436 nm is >3 indicating the fraction of light absorbed is >0.999.

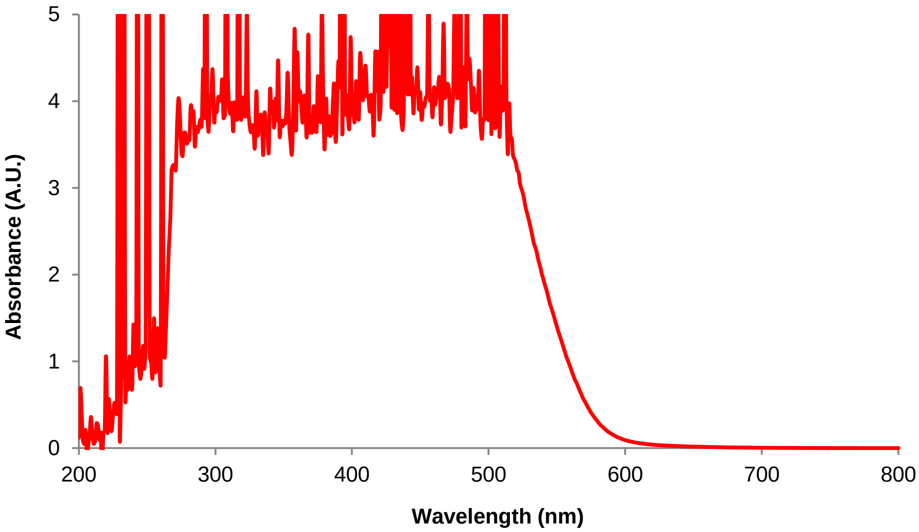

Figure 14. Absorbance of a 2.5×10^{-3} M solution of $Ru(bpy)_3Cl_2$ in DMF.

Stern–Volmer quenching rate data:

Nicewicz and MacMillan report a Stern–Volmer constant ($K_{SV} = k_q\tau_0$) of 10 M^{-1} for the enamine formed *in situ* and found no quenching of $Ru(bpy)_3^{2+}$ by any of the other reagents.[12] The k_q for the enamine was derived from this Stern–Volmer constant ($k_q = 1.1 \times 10^7\ M^{-1}\ s^{-1}$). The lifetime measurement for $Ru(bpy)_3Cl_2$ in DMF (912 ns) was previously reported.[11]

The concentration of the enamine at initial reaction conditions was estimated by ^1H NMR. In an NMR tube were combined 75 µL octanal, 41 µL diethyl bromomalonate, 15.4 mg organocatalyst **12**•OTf, 45 µL 2,6-lutidine and 0.50 mL DMF. The ^1H NMR showed a 3.2:1 ratio of organocatalyst (**12**) to enamine (**14**). This corresponds to a 0.018 M concentration of enamine under the standard reaction conditions (0.031 mmol enamine in a total volume of 1.76 mL).

Quenching fraction calculation:

$$Q = \frac{k_{q,\mathbf{14}}[\mathbf{14}]}{\tau_0^{-1} + k_{q,\mathbf{14}}[\mathbf{14}]}$$

$$= \frac{1.1 \times 10^7\ M^{-1}s^{-1}[0.018\ M]}{\frac{1}{9.12 \times 10^{-7}\ s} + 1.1 \times 10^7\ M^{-1}s^{-1}[0.018\ M]}$$

$$= 0.15$$

The equilibrium constant, K_{eq}, for enamine formation was calculated using eq 10.

$$K_{eq} = \frac{[\mathbf{14}][H_2O]}{[\mathbf{11}][\mathbf{12}]} \quad (10)$$

$$= \frac{[0.018\ M][0.018\ M]}{[0.71\ M][0.056\ M]}$$

$$= 8.1 \times 10^{-3}$$

Simple quenching experiment:

The quenching fraction was determined by eq 7 and averaged over the first 90 s of the reaction. For the α-alkylation, the luminescence intensity (detected at λ = 600 nm, 10.0 nm slit width) without the organocatalyst quencher **12** (I_0) was measured by combining 240 µL (2 equiv) octanal, 230 µL (1 equiv) diethyl bromomalonate, 160 µL (1.8 equiv) 2,6-lutidine, 39.2 mg (0.2 equiv) 2,6-lutidine triflate salt, 2.9 mg (0.5 mol%) $Ru(bpy)_3Cl_2{\bullet}6H_2O$, and 1.5 mL (0.5 M) DMF in a cuvette. The intensity with the quencher (I) was recorded by combining the above solution with 25.8 mg (0.2 equiv) of **12**. The triflic acid is necessary in the I_0 measurement to obtain accurate results.

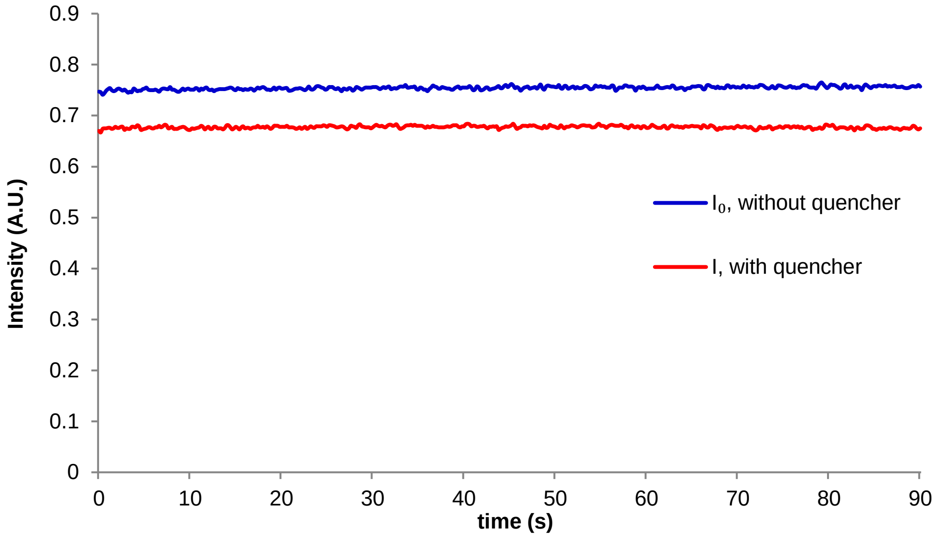

Figure 15. Phosphorescence intensity of the reaction over 90 s with (red line) and without (blue line) organocatalyst **12**.

For this reaction, Q = 0.10.

Chain length calculation:

Chain length values calculated in this paper are a lower limit approximation of the actual chain lengths and were calculated using eq 8, where Q was calculated either through the Stern–Volmer analysis or the simple quenching experiment.

For the Stern–Volmer analysis, the chain length was calculated to be 120; for the simple quenching experiment, the chain length was 180.

Light/dark experiment:

A Schlenk tube was equipped with a stir bar and charged with 300 µL octanal (1.9 mmol), 165 µL diethyl bromomalonate (0.97 mmol), 3.7 mg $Ru(bpy)_3Cl_2$•$6H_2O$ (0.0049 mmol), 62.0 mg of **12**•OTf (0.19 mmol), 230 µL 2,6-lutidine (2.0 mmol), 40 µL trimethyl(phenyl)silane (0.23 mmol), and 2.0 mL (0.50 M) DMF. The reaction vessel was degassed by three cycles of freeze-pump-thaw and backfilled with N_2. The reaction was alternatively irradiated with a 20 W CFL bulb and kept in the dark in twenty minute intervals. Aliquots were taken at the start and after each interval, passed through a silica plug, and diluted with $CDCl_3$. Yields of **13** were determined by ^{1}H NMR and based on trimethyl(phenyl)silane as an internal standard.

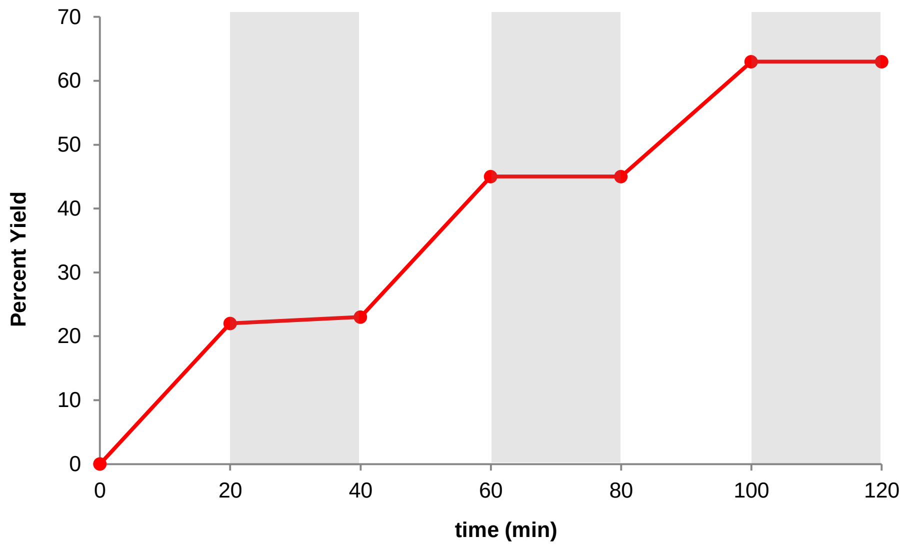

Figure 16. Light/dark experiment.

VI. *N,N*-Dimethyl-*p*-toluidine studies

Stern–Volmer quenching rate data:

Samples were prepared by adding solutions of photocatalyst, *N*,*N*-dimethyl-*p*-toluidine, and DMF to obtain a total volume of 2.0 mL. The cuvette was sealed with a septum and parafilm, and then sparged for 15 min with N_2. The concentration of $Ru(bpy)_3Cl_2$ was 5.0×10^{-5} M. Samples were irradiated at 455 nm, and emission was detected at 600 nm.

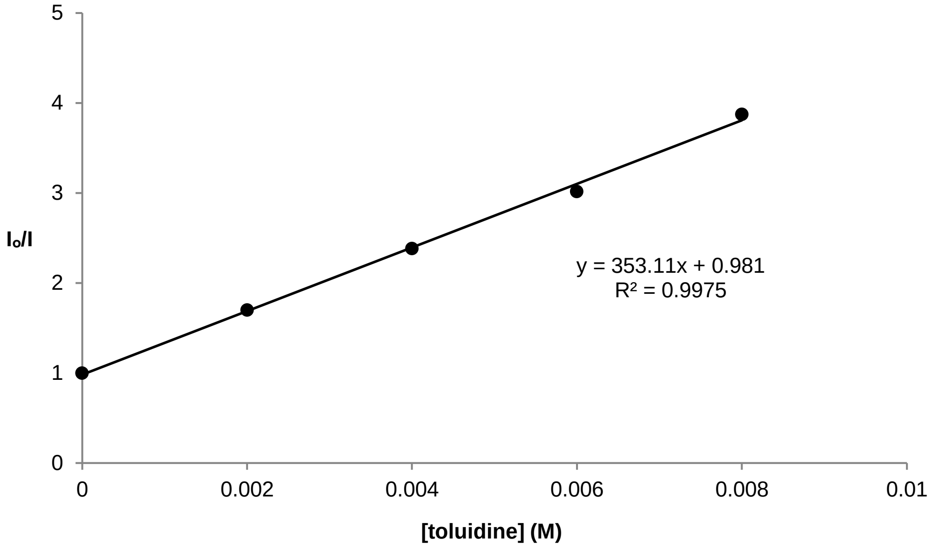

Figure 17. Stern–Volmer quenching of $Ru(bpy)_3Cl_2$ and *N*,*N*-dimethyl-*p*-toluidine. For the amine, $k_q = 3.9 \times 10^8\ M^{-1}\ s^{-1}$.

Time course experiments:

For the standard conditions, a Schlenk tube was equipped with a stir bar and charged with 200 µL octanal (1.3 mmol), 110 µL diethyl bromomalonate (0.65 mmol), 2.4 mg $Ru(bpy)_3Cl_2$•$6H_2O$ (0.0032 mmol), 41.3 mg of **12**•OTf (0.13 mmol), 150 µL 2,6-lutidine (1.3 mmol), 29.5 mg phenanthrene (0.16 mmol), and 1.3 mL (0.50 M) DMF. The reaction vessel was degassed by three cycles of freeze-pump-thaw and backfilled with N_2. The reaction was alternatively irradiated with a 20 W CFL bulb and kept in the dark in ten minute intervals. Aliquots were taken at the start and after each interval, passed through a silica plug, and concentrated. Yields of **13** were determined by ^{1}H NMR and based on phenanthrene as an internal standard.

For the addition of exogenous quencher, a Schlenk tube was equipped with a stir bar and charged with 200 µL octanal (1.3 mmol), 110 µL diethyl bromomalonate (0.65 mmol), 2.4 mg $Ru(bpy)_3Cl_2$•$6H_2O$ (0.0032 mmol), 41.4 mg of **12**•OTf (0.13 mmol), 150 µL 2,6-lutidine (1.3 mmol), 0.40 mg *N*,*N*-dimethyl-*p*-toluidine (0.0030 mmol), 33.6 mg phenanthrene (0.19 mmol), and 1.3 mL (0.50 M) DMF. The reaction vessel was degassed by three cycles of freeze-pump-thaw and backfilled with N_2. The reaction was alternatively irradiated with a 20 W CFL bulb and kept in the dark in ten minute intervals. Aliquots were taken at the start and after each interval, passed through a silica plug, and concentrated. Yields of **13** were determined by ^{1}H NMR and based on phenanthrene as an internal standard.

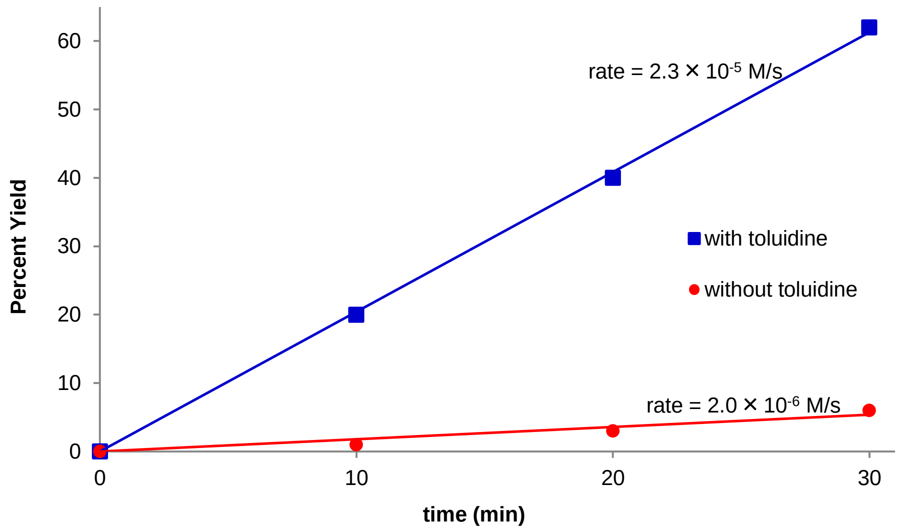

Figure 18. Time course of the reaction with (blue line) and without (red line) *N*,*N*-dimethyl-*p*-toluidine.

Experiments without $Ru(bpy)_3Cl_2$:

Scheme 6. Asymmetric α-alkylation of octanal in the absence of $Ru(bpy)_3Cl_2$.

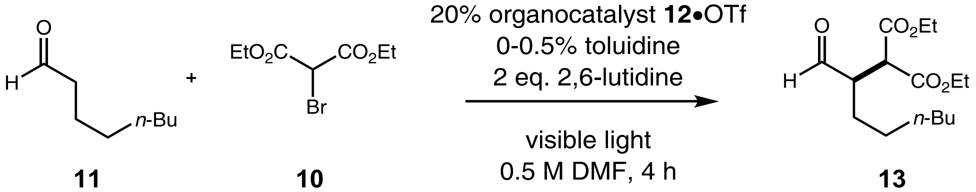

11 10 13

A Schlenk tube was equipped with a stir bar and charged with 200 µL octanal (1.3 mmol), 110 µL diethyl bromomalonate (0.65 mmol), 41.3 mg of **12**•OTf (0.13 mmol), 150 µL 2,6-lutidine (1.3 mmol), 26.0 mg phenanthrene (0.15 mmol), and 1.3 mL (0.50 M) DMF. The reaction vessel was degassed by three cycles of freeze-pump-thaw and backfilled with N_2. The reaction was irradiated with a 20 W CFL bulb for 1 h, then passed through a silica plug and concentrated. The reaction yielded 13% of **13** as determined by ^{1}H NMR using phenanthrene as an internal standard.

A Schlenk tube was equipped with a stir bar and charged with 200 µL octanal (1.3 mmol), 110 µL diethyl bromomalonate (0.65 mmol), 41.3 mg of **12**•OTf (0.13 mmol), 150 µL 2,6-lutidine (1.3 mmol), 0.40 mg *N*,*N*-dimethyltoluidine (0.0030 mmol), 30.7 mg phenanthrene (0.17 mmol), and 1.3 mL (0.50 M) DMF. The reaction vessel was degassed by three cycles of freeze-pump-thaw and backfilled with N_2. The reaction was irradiated with a 20 W CFL bulb for 1 h, then passed through a silica plug and concentrated. The reaction yielded 11% of **13** as determined by ^{1}H NMR using phenanthrene as an internal standard.

Determination of enantioselectivity:

Scheme 7. Conversion of **10** into the corresponding hydrazone.

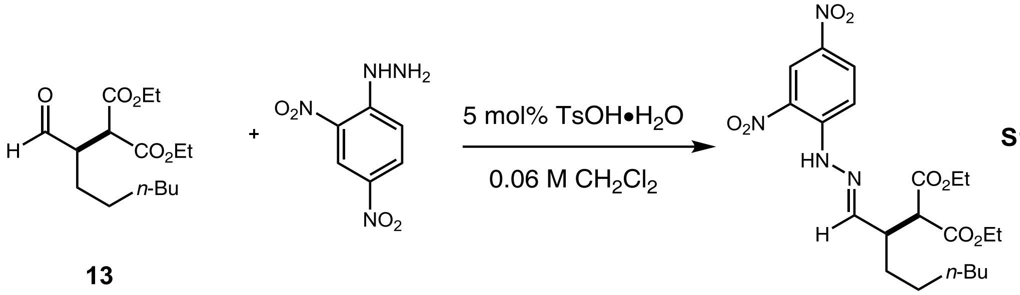

The enantiomeric excess of the product was determined by converting **13** into the corresponding hydrazone (**S1**) using 2,4-dinitrophenylhydrazine. A vial was charged with **13** (0.12 mmol, 1 equiv), 2,4-dinitrophenylhydrazine (0.12 mmol, 1 equiv), *p*-toluenesulfonic acid monohydrate (0.0060 mmol, 5 mol%), and 2.0 mL (0.06 M) CH_2Cl_2. The reaction was stirred for 2.5 h and then was purified *via* silica gel chromatography. Enantiomeric excesses were determined by supercritical fluid chromatography (SFC) on a TharSFC investigator instrument equipped with a Waters 2996 photodiode array detector using a Daicel CHIRALCEL®OJ-H chiral column, 5-50% MeOH gradient over 15 min, λ = 345 nm, t_1 = 4.2 min, t_2 = 4.9 min. Racemic **13** was synthesized using morpholine in place of **12**•OTf.

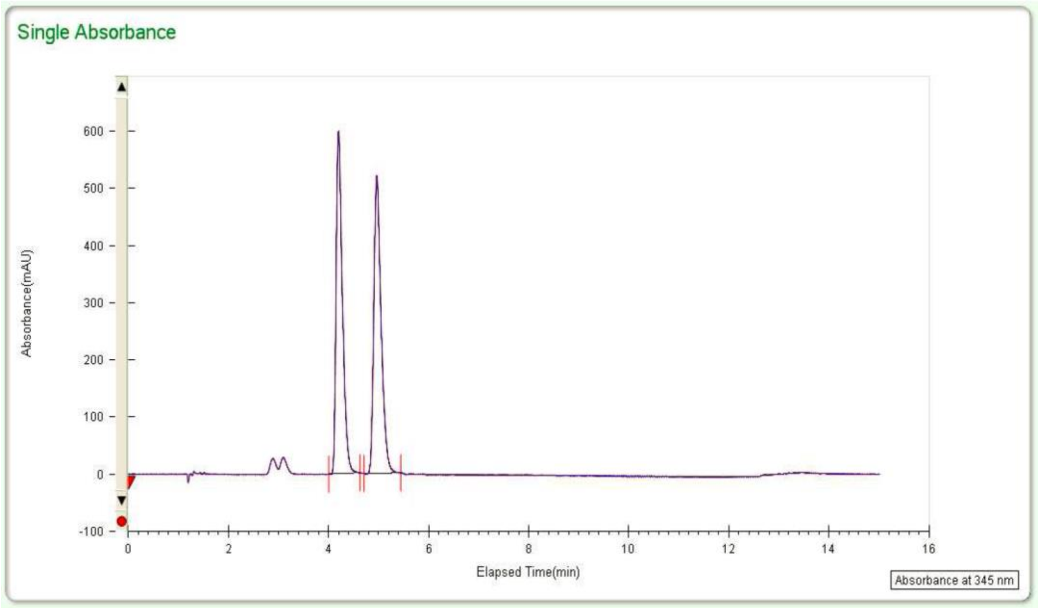

Peak Info

Peak No	% Area	Area	RT (min)	Height (mV)
1	49.9213	5231.9348	4.2	598.8074
2	50.0787	5248.4205	4.96	520.3611
Total:	100	10480.3553		

Figure 19. SFC chromatogram of racemic hydrazone **S1**.

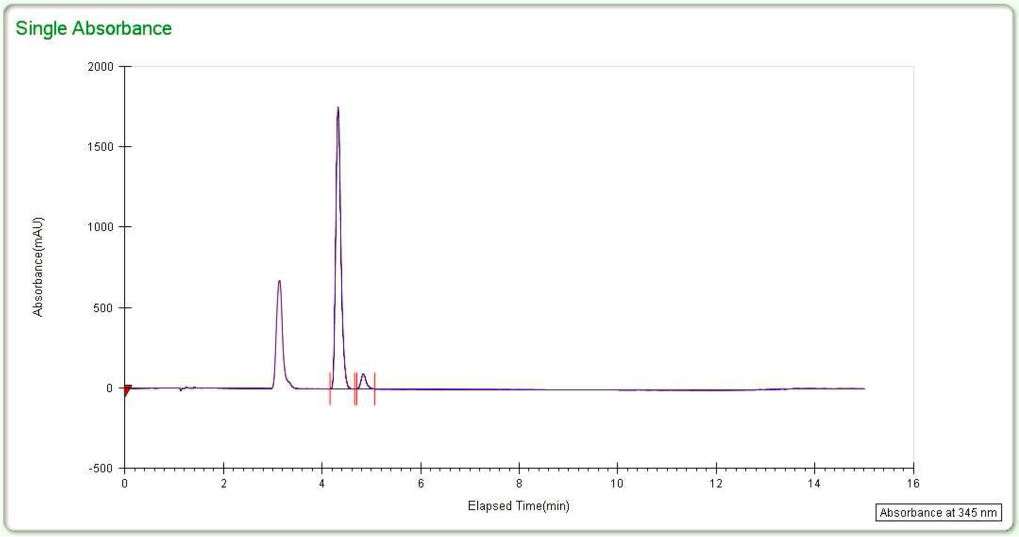

Peak Info

Peak No	% Area	Area	RT (min)	Height (mV)
1	95.0561	12874.3714	4.32	1753.6597
2	4.9439	669.6068	4.84	92.4535
Total:	100	13543.9782		

Figure 20. SFC chromatogram of **S1** (90% ee) from **13** that was synthesized using the standard conditions reported by MacMillan.[12]

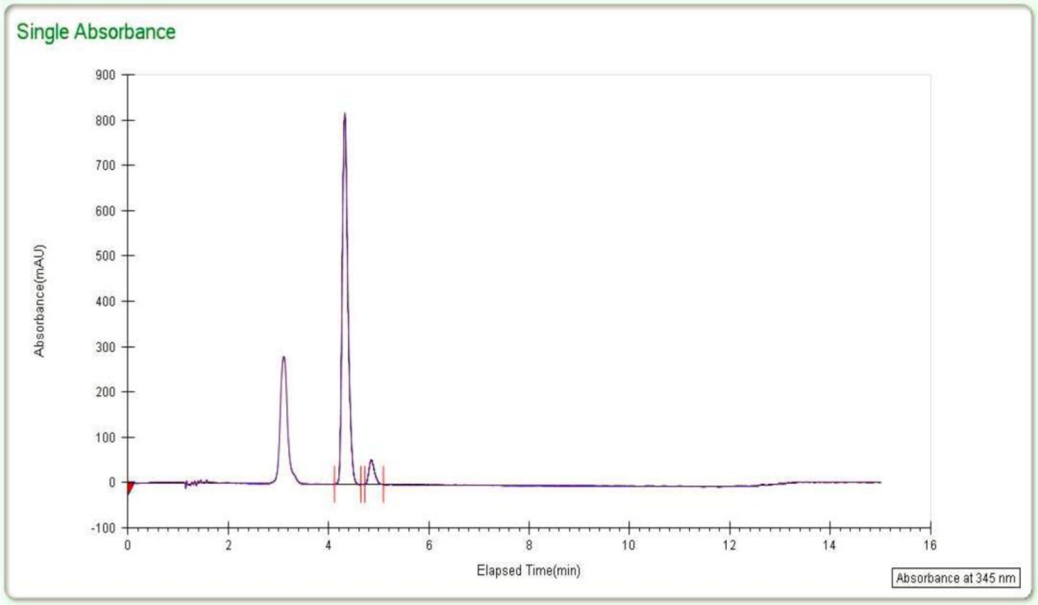

Peak Info

Peak No	% Area	Area	RT (min)	Height (mV)
1	93.885	6431.0714	4.32	817.7973
2	6.115	418.8743	4.85	54.1336
Total:	100	6849.9457		

Figure 21. SFC chromatogram of **S1** (88% ee) from **13** that was synthesized using the method with *N*,*N*-dimethyl-*p*-toluidine.

VII. References

[1] Pagnorn, A. B.; Giardello, M. A.; Grubbs, R. H.; Rosen, R. K.; Timmers, F. J. *Organometallics* **1996**, *15*, 1518–1520.
[2] Schultz, D. M.; Sawicki, J. W.; Yoon, T. P. *Beilstein J. Org. Chem.* **2015**, *11*, 61–65.
[3] Wang, L. –C.; Jang, H. –Y.; Roh, Y.; Lynch, V.; Schultz, A. J.; Wang, X.; Krische, M. J. *J. Am. Chem. Soc.* **2002**, *124*, 9448–9453.
[4] Graham, T. H.; Horning, B. D.; MacMillan, D. W. C. *Org. Syn.* **2011**, *88*, 42–54.
[5] Hatchard, C. G.; Parker, C. A. *Proc. Roy. Soc. (London)* **1956**, *A235*, 518–536.
[6] a) Kuhn, H. J.; Braslavsky, S. E.; Schmidt, R. *Pure Appl. Chem.* **2004**, *76*, 2105–2146. b) Monalti, M. *et. al.* Chemical Actinometry. *Handbook of Photochemistry*, 3rd Ed; Taylor & Francis Group, LLC. Boca Raton, FL, **2006**, 601–616.
[7] Lin, S.; Ischay, M. A.; Fry, C. G.; Yoon, T. P. *J. Am. Chem. Soc.* **2011**, *133*, 19350–19353.
[8] Barqawi, K. R.; Akasheh, T. S.; Beaumont, P. C.; Parsons, B. J.; Phillips, G. O. *J. Phys. Chem.* **1988**, *92*, 291–294.
[9] For a χ for oxygen in CH_2Cl_2, see: Shirono, K.; Morimatsu, T.; Takemura, F. *J. Chem. Eng. Data* **2008**, *53*, 1867–1871.
[10] Ischay, M. A.; Anzovino, M. E.; Du, J.; Yoon, T. P. *J. Am. Chem. Soc.* **2008**, *130*, 12886–12887.
[11] Caspar, J. V.; Meyer, T. J. *J. Am. Chem. Soc.* **1983**, *105*, 5583–5590.
[12] Nicewicz, D.; MacMillan, D. W. C. *Science*, **2008**, *322*, 77–80.
